# A comprehensive review of viscoelastic polymer flooding in sandstone and carbonate rocks

**DOI:** 10.1038/s41598-023-44896-9

**Published:** 2023-10-17

**Authors:** Mursal Zeynalli, Muhammad Mushtaq, Emad W. Al-Shalabi, Umar Alfazazi, Anas M. Hassan, Waleed AlAmeri

**Affiliations:** Chemical and Petroleum Engineering Department, Khalifa University of Science and Technology, SAN Campus, Abu Dhabi, UAE

**Keywords:** Petrology, Polymer chemistry

## Abstract

Polymer flooding is a proven chemical Enhanced Oil Recovery (cEOR) method that boosts oil production beyond waterflooding. Thorough theoretical and practical knowledge has been obtained for this technique through numerous experimental, simulation, and field works. According to the conventional belief, this technique improves macroscopic sweep efficiency due to high polymer viscosity by producing moveable oil that remains unswept after secondary recovery. However, recent studies show that in addition to viscosity, polymer viscoelasticity can be effectively utilized to increase oil recovery by mobilizing residual oil and improving microscopic displacement efficiency in addition to macroscopic sweep efficiency. The polymer flooding is frequently implemented in sandstones with limited application in carbonates. This limitation is associated with extreme reservoir conditions, such as high concentrations of monovalent and divalent ions in the formation brine and ultimate reservoir temperatures. Other complications include the high heterogeneity of tight carbonates and their mixed-to-oil wettability. To overcome the challenges related to severe reservoir conditions, novel polymers have been introduced. These new polymers have unique monomers protecting them from chemical and thermal degradations. Monomers, such as NVP (N-vinylpyrrolidone) and ATBS (2-acrylamido-2-methylpropane sulfonic acid), enhance the chemical resistance of polymers against hydrolysis, mitigating the risk of viscosity reduction or precipitation in challenging reservoir conditions. However, the viscoelasticity of these novel polymers and their corresponding impact on microscopic displacement efficiency are not well established and require further investigation in this area. In this study, we comprehensively review recent works on viscoelastic polymer flow under various reservoir conditions, including carbonates and sandstones. In addition, the paper defines various mechanisms underlying incremental oil recovery by viscoelastic polymers and extensively describes the means of controlling and improving their viscoelasticity. Furthermore, the polymer screening studies for harsh reservoir conditions are also included. Finally, the impact of viscoelastic synthetic polymers on oil mobilization, the difficulties faced during this cEOR process, and the list of field applications in carbonates and sandstones can also be found in our work. This paper may serve as a guide for commencing or performing laboratory- and field-scale projects related to viscoelastic polymer flooding.

## Introduction

The traditional waterflooding method has long been associated with suboptimal oil recovery rates and the early onset of water production. The imperative here is to mitigate excessive water cut in order to achieve the necessary uniform sweep and bolster hydrocarbon extraction; otherwise, there is a significant jeopardy of substantial reductions in oil recovery^1–3^. This issue extends beyond purely technical concerns, as the overproduction of water during the oil recovery process carries detrimental financial and environmental consequences^4^. Premature water production can abbreviate the operational lifespan of wells and elevate the expenses associated with safely disposing of the generated water, issues that weigh heavily on the minds of oil producers.

In contrast, the implementation of polymer flooding offers a viable solution to address these challenges. By effectively managing excessive water production, polymer flooding maximizes oil recovery and significantly augments the return on investment for oil companies^5^. Essentially, the polymer flooding technique is employed to manage the mobility and conformance of the injected fluid, thereby enhancing the macroscopic sweep efficiency (${E}_{v}$)^6^ by producing oil bypassed during waterflooding. Mobility control is particularly crucial when the viscosity of the oil significantly surpasses that of the driving fluid. Viscous fingering, a phenomenon detrimental during secondary and tertiary recovery phases due to its tendency to create uneven flow profiles and cause early water breakthroughs, can be mitigated through polymer flooding. This method works by reducing the viscosity contrast between the oil and the injected fluid, ultimately improving areal sweep efficiency.

**Figure 1:**
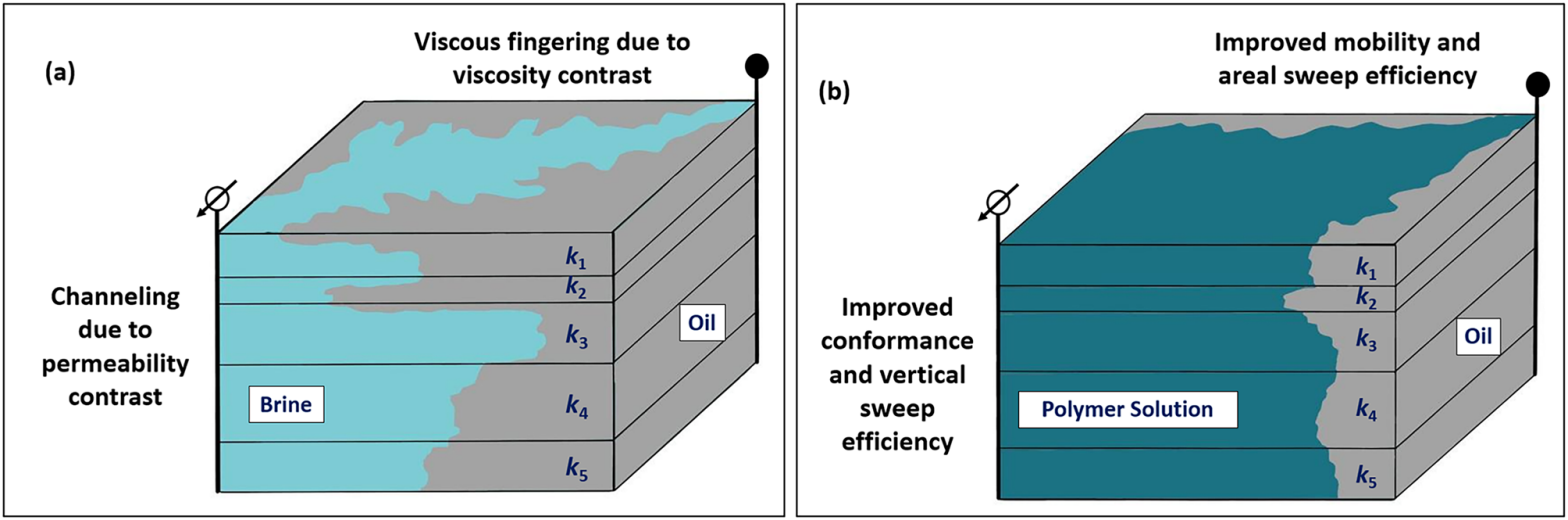
Schematic of macroscopic sweep efficiency enhancements by polymer flooding: (**a**) viscous fingering and channeling in heterogeneous formations (*k*_3_ > *k*_4_ > *k*_5_ > *k*_1_ > *k*_2_) significantly reduce sweep efficiency during waterflooding, (**b**) injecting polymer solution can improve mobility and conformance of injectant and increase oil recovery efficiency.

Furthermore, conformance control boosts vertical sweep efficiency by impeding channeling through various reservoir layers. Typically, channeling arises from the uneven permeability distributions found in heterogeneous formations. However, it can also be attributed to various other factors, including reservoir compartmentalization and geological conditions. Field operations, such as inadequate well spacing, unsuccessful completion and stimulation practices, can exacerbate the adverse effects of low vertical sweep efficiency^7^. Water injection into such reservoirs during secondary oil recovery results in redundant water outflow from highly permeable zones, leaving considerable volumes of mobile oil in low-permeable layers. As a solution, utilizing polymers in the aqueous phase during the tertiary recovery ensures a minimal change in the relative permeability to oil while reducing the relative permeability of water. This approach, known as disproportionate permeability reduction (DPR), enhances vertical sweep efficiency by diverting the driving fluid from highly permeable "thief zones" to tight areas of the reservoir^8^. It was found that polymer adsorption onto rock surfaces might be the primary mechanism causing DPR^9,10^. Significantly, disproportionate permeability reduction is influenced by the characteristics of both the rock and the fluid, with a more substantial effect seen in water-wet reservoirs as opposed to oil-wet porous media^11^. Moreover, additional enhancement in conformance control was particularly observed with viscoelastic polymers, which can dramatically reduce the relative permeability of an aqueous phase due to their elasticity^12^. The considerable decrease in aqueous phase relative permeability caused by viscoelastic polymers is related to their ability to create stable and temporary bridges within porous media, leveraging their elastic properties. When subjected to flow, these polymers can deform and stretch elastically, forming resilient structures that may span pore throats and constrict fluid pathways. This elasticity allows the polymer bridges to effectively impede the movement of the aqueous phase, thereby reducing its relative permeability^13,14^. Figure 1 depicts the increase in macroscopic sweep efficiency in a heterogeneous formation (*k*_3_ > *k*_*4*_ > *k*_5_ > *k*_1_ > *k*_2_, where *k* is formation permeability) by controlling the mobility and conformance of an injectant through the polymer flooding technique.

Moreover, it is essential to discuss polymer gels in the context of conformance control, as they are among the most critical agents for achieving conformance control. The process involves injecting a mixture known as a "gelant" into the target area, which then solidifies into a semi-solid gel after a predetermined period. Any chemical capable of acting as a plugging agent when introduced into the reservoir is considered a "conformance agent." Water-blocking polymer gels are widely employed as common conformance agents in oil recovery applications. These polymer gels primarily serve conformance control by obstructing high-permeability regions and redirecting injected water from areas with high permeability to those with lower permeability in the reservoir. They also contribute to profile modification and water shutoff. This results in improved sweep efficiency and enhanced oil displacement, which are critical processes for boosting oil recovery in heterogeneous oil reservoirs^15^. In summary, polymer gels emerge as highly effective agents for achieving conformance control, enhancing sweep efficiency, and reducing excessive water production by mitigating reservoir heterogeneity. Among these gels, widely utilized polyacrylamide polymer gels can be categorized into three main types based on their compositions and application scenarios: in-situ monomer-based gels, in-situ polymer-based gels, and preformed particle gels. The first category, in-situ monomer gels, primarily employs acrylamide as the main component and was initially developed for water shutoff. The second category includes metal-cross-linked polyacrylamide gels and organic-cross-linked polyacrylamide gels, both representing conventional in-situ polymer gels. In addition, there are preformed gels such as preformed particle gels, pH-sensitive microgels, and micro- and nano-gels^1^. These diverse types of gels offer various solutions for improving conformance control in reservoir management.

On the other hand, microscopic displacement efficiency (${E}_{d}$) is a more pore-scale parameter related to residual oil saturation^16^. Recent works have shown that viscoelastic polymers can improve oil recovery beyond that of inelastic polymers^16–20^. It is related to the ability of viscoelastic polymers to mobilize residual oil and increase microscopic displacement efficiency, in addition to macroscopic sweep efficiency. It is believed that the oil entrapped in porous media due to high capillary forces, rock configuration, or rock attraction can be produced by viscoelastic polymers by various mechanisms, such as stripping off, dragging, and pulling the oil molecules to the pore channels^20^. Improving ${E}_{d}$ by lowering residual oil saturation will be discussed in more detail later in the paper.

In general, polymer viscoelastic behavior and the oil recovery mechanisms of such polymers have been described in the literature^11,21,22^. This study provides a comprehensive and critical summary of recent advancements in viscoelastic polymer flooding applications in sandstones and carbonates. The paper can be used as a reference for initiating a viscoelastic polymer flooding project. Moreover, the provided recommendations and the literature gaps reported here can serve as a guide for performing future research in this area.

## Polymer flooding under extreme reservoir conditions

This section represents the recent advancements in polymer flooding under harsh reservoir conditions. Accordingly, specially tailored polymers based on hydrolyzed polyacrylamide (HPAM) have been developed to resist high temperatures and high salinities in carbonate reservoirs. The classification and description of various novel polymers and screening studies evaluating their performance under extreme conditions are reviewed in the coming subsections.

### Classification of EOR polymers

The main categories of EOR polymers include biopolymers and synthetic polymers. The advantages and shortcomings of various biopolymers and synthetic polymers are listed in Table 1. Biopolymers, while able to withstand higher salinity and temperature levels compared to synthetic polyacrylamides, suffer from limited injectivity and susceptibility to biological degradation^23–26^. On the other hand, synthetic polymers, particularly HPAM, are preferred for field applications due to their cost-effectiveness, ease of on-site manufacture, and better control over molecular weight distribution^27–30^. Another distinction between biopolymers and synthetic polymers lies in the flexible random-coil structure of the latter. Under favorable conditions, this structure allows synthetic polymers to exhibit dilatant behavior in porous media, whereas the same conditions do not lead to the viscoelasticity of biopolymers in such media. Dilatant behavior, or shear-thickening, causes viscoelastic synthetic polymers to experience a significant increase in viscosity at high shear rates. This phenomenon can ultimately result in enhanced sweep efficiency and improved oil recovery^17,31^.

**Table 1:** A comparison of biopolymers and synthetic polymers.

Features	Biopolymers	Synthetic Polymers
Temperature Tolerance	Biopolymers have an advantage in terms of thermal stability. In particular, scleroglucan and diutan gum can resist temperatures up to 150 °C^266^. Compared with scleroglucan, xanthan is not stable at temperatures above 90 °C^215,267^	HPAM is susceptible to thermal degradation when exposed to temperatures exceeding 75 °C^35,268^. Application of novel polymers with specific monomers, such as NVP or ATBS, can boost the thermal stability to above 100 °C^122^
Salinity Tolerance	Another advantage of biopolymers is their salinity tolerance; most biopolymers can withstand salinities greater than 150 g/L^269–272^	HPAM can precipitate at high salinities, particularly when the multivalent ion concentration exceeds 0.2 g/L^35^. HAP and other polymers incorporating NVP or ATBS can tolerate highly saline brines with TDS more than 160 g/L^44,53^
Shear Tolerance	Stable helical conformation of most biopolymers helps them in resisting shear degradation in porous media^8,273^	PAM and HPAM polymers are quite susceptible to mechanical degradation, especially when injected at high M_w_^215^. NVP- or ATBS-based copolymers may have better stability^55,274^
Tolerance to Biological Degradation	One notable drawback of biopolymers is their susceptibility to microbial degradation^26,273,275^	Synthetic polymers have an advantage to withstand biological degradation^55^
Injectivity	Another disadvantage of most biopolymers is their poor injectivity^55^. However, some studies provided promising injectivity results for scleroglucan^274^	Poor injectivity is observed when high M_w_ HPAM or HAP is injected into tight rock^140^. Copolymers may have better injectivity^40,276^
Manufacturing Cost	The manufacturing cost is 12 USD/kg for xanthan, 50 USD/kg for scleroglucan, and 8.5–11 USD/kg for schizophyllan^273,277^	Conventional HPAM polymers are cheaper than biopolymers, and the cost of manufacturing ranges from 5 to 11 USD/kg^273^. However, incorporating NVP or ATBS monomers in the polymer chain can considerably increase its cost^38,40,55^
Environmental Toxicity	Biopolymers are non-toxic^55^	Unlike biopolymers, HPAM can release neurotoxins and potential carcinogens^278^
Optimal pH Range	The optimal pH range for xanthan is 7–8^279^, while it varies from 12–13 for scleroglucan^280^	Neutral pH is required to avoid rapid hydrolysis of HPAM in porous media^35,215^. Basic pH range is recommended to stabilize the hydrolysis rate of polymers with ATBS content^281^
Adsorption	The rigid xanthan molecules adhere flatly to the pore wall, and the hydraulic radius of the porous medium is not substantially affected^282^	The adsorption-layer thickness of synthetic polymers may depend on permeability, molecular weight, rock mineralogy, wettability, and water chemistry^33,215,220^
Flow Behavior	Most biopolymers exhibit shear-thinning behavior in porous media^75^	Synthetic polymers usually show Newtonian and shear-thickening regimes in low and high shear rate regions, respectively

**Figure 2:**
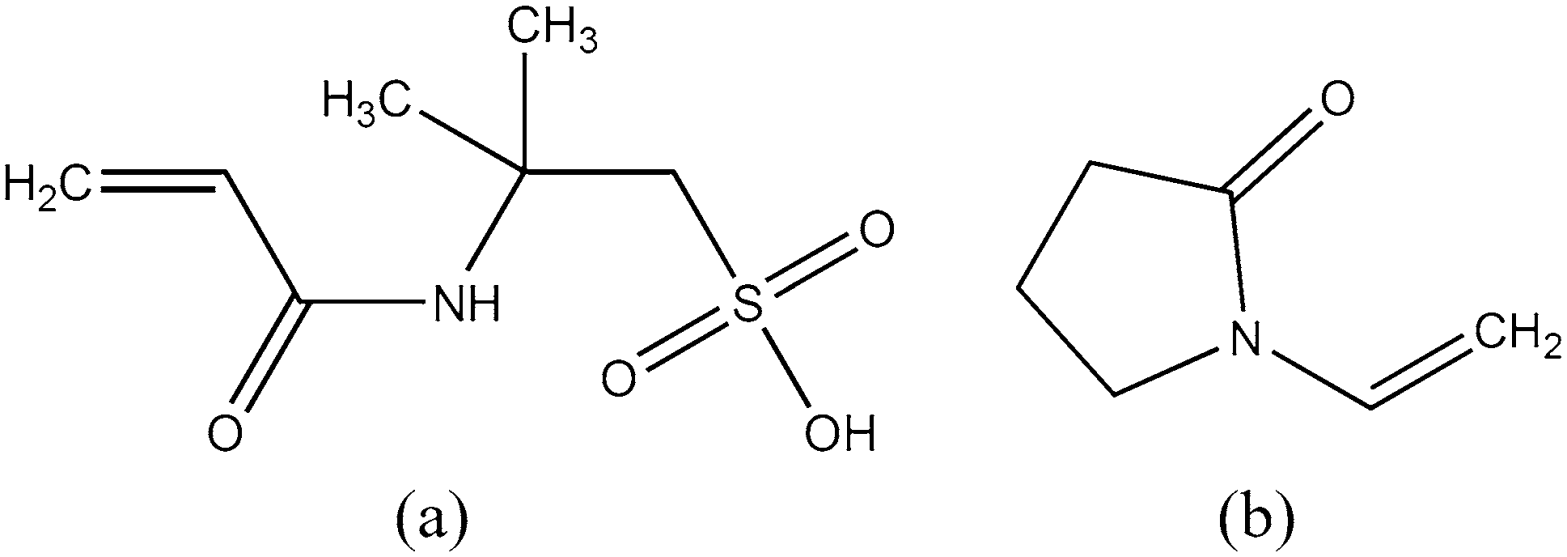
Chemical structures of (**a**) Acrylamido Tertiobutyl Sulfonate (ATBS) and (**b**) N-vinylpyrrolidone (NVP).

Despite all favorable attributes, HPAM-based polymers are not stable at high temperatures in the presence of high salinity with divalent cations. According to Seright et al.^32^, acrylamide-based polymers undergo degradation through three different mechanisms when exposed to high temperatures. The first mechanism involves amide/side-group hydrolysis, followed by precipitation with divalent cations. The second mechanism involves the reaction with dissolved oxygen, leading to the cleavage of the C–C backbone. The third mechanism involves the breakage of the C–C backbone, which occurs through some other processes that do not require the presence of dissolved oxygen. Currently, the top focus in the HPAM application is the development of novel HPAM-based polymers that can withstand extreme reservoir conditions of high salinity and high temperature^33,34^. The new polymers with expanded application envelopes incorporate various monomers, enhancing their stability under harsh conditions. These monomers include Sodium Acrylamido Tertiobutyl Sulfonate (ATBS, also known as 2-Acrylamido-2-Methylpropane-Sulfonate (AMPS)) and N-vinylpyrrolidone (NVP), structures are shown in Fig. 2. ATBS are anionic monomers that can resist precipitation and cation shielding. Therefore, they may improve the calcium tolerance of a copolymer^35,36^. On the other hand, NVP nonionic monomers are used to stabilize acrylamide groups against thermal hydrolysis and can increase the temperature resistance of terpolymers to 120 °C^37^. It is also important that the concentration of NVP in the polymer chain should be between 35 and 50 mol% in the presence of divalent ions for sufficient stability at high temperatures^38^. Concerning ATBS, a sulfonation degree of around 32 mol% (60 wt%) is typical for most commercial sulfonated polymers, including VHM (32 mol%), SUPERPUSHER SAV55 (35 mol%), and THERMOASSOCIATIF polymers (around 32 mol%). A very stable polymer, SAV10, may have a sulfonation degree higher than 45 mol%^39^.

However, it is essential to consider that NVP has certain drawbacks, including a reduction in polymer molecular weight, increased polymer costs, and a negative impact on injectivity in low-permeability tight carbonates^40^. As an alternative, incorporating the more cost-effective ATBS, which has a less detrimental effect on polymer molecular weight compared to NVP, may enhance the stability and performance of a polymer to some extent^33^. While ATBS offers certain advantages, it is noteworthy that polymers containing ATBS may experience a decline in performance at elevated temperatures due to their increased susceptibility to hydrolysis in such conditions. On the other hand, the NVP monomers may constrain acrylamide hydrolysis through the so-called neighboring effect^34,41^.

#### Hydrophobically associated polymers

It is also crucial to describe another group of polymers known for their resilience under harsh conditions. Hydrophobically Associated Polymers (HAPs) represent a distinct type of polymer derived from HPAM, setting them apart from conventional HPAM and its other derivatives. The introduction of a limited number of hydrophobic groups, typically consisting of 8–18 carbon atoms, into the hydrophilic polyacrylamide chain can induce significant alterations in HPAM behavior. The polar and non-polar parts of macromolecules are rearranged in aqueous solutions, leading to the formation of hydrophobic associations between the incorporated hydrophobic groups. These modified HPAM polymers are called associative polymers, and they show an association between hydrophobic groups within a macromolecule and between hydrophobic groups in neighboring macromolecules. These polymers have unique structural aspects, and their properties highly depend on the structure, salinity, temperature, and especially concentration. The viscosity of these polymers can increase significantly after reaching a specific concentration, called the critical association concentration (CAC). The CAC represents the concentration beyond which intermolecular associations with the polymer chains initiate, serving as the defining threshold concentration that characterizes the typical behavior of these polymers^42^. At CAC, the hydrophobic groups form intramolecular and intermolecular associations, resulting in three-dimensional networks that increase the viscosity of the polymer solution at lower concentrations compared to HPAM^43^.

Studies have shown that HAPs have greater resistance to changes in salinity and have increased hydrophobic interactions in the presence of divalent ions, rendering them a promising option for high-salinity carbonate reservoirs^44^. Researchers have reported that associative polymer injection in porous media results in significantly higher resistance factors compared to traditional non-associative polymers^45–47^. This is because the restricted space during flow in porous media leads to higher hydrophobic interactions even at lower concentrations than in bulk^48,49^. The literature has also explored the viscoelastic properties of associative polymers employing a capillary breakup extensional rheometer (CaBER)^12^. These reports suggest that associative polymers may exhibit more pronounced viscoelastic behavior with higher extensional relaxation times and extensional viscosities compared to conventional HPAM polymers. The enhanced viscoelasticity becomes particularly evident at polymer concentrations exceeding the CAC. Azad and co-authors^48^ shared that increased extensional viscosity leads to greater polymer stretching, resulting in higher retention and permeability reduction. However, significant permeability reduction during associative polymer flooding is contingent on hydrophobic association aided by intermolecular effects. Hence, the conversion of the intermolecular network to an intramolecular network at low polymer concentrations (below CAC) or under high flux rates can restrict both permeability reduction and the resistance factor values achieved by associative polymers. Furthermore, recent studies have demonstrated that associative polymers can reduce residual oil saturation in porous media by promoting hydrophobic interactions between the polymer and crude oil molecules^50,51^.

Although hydrophobically associated polymers have several advantages, they also have some drawbacks. Several researchers have reported very high RF values, which can cause plugging and injectivity issues, particularly in tight reservoirs^46^. Moreover, the intrinsic hydrophobic nature of these polymers may create emulsions with crude oil, which can create difficulties during processing in subsequent stages^51^. To improve the injectivity of associative polymers in low-permeability rocks, shear degradation is recommended, as it reduces particle size distribution and enhances polymer transport^52^.

### Polymer screening studies for harsh conditions

Polymers in porous media under extreme conditions have been extensively studied^23–26^. However, our primary focus will remain on the polymer screening studies evaluating novel HPAM-based polymers. For instance, Levitt and Pope^35^ investigated commercial polymers for thermal, chemical, and surfactant compatibility and reported that the polyacrylamide solutions were stable at elevated temperatures only when the hardness of the brine was less than 200 ppm. They proposed that in the presence of a larger concentration of divalent ions and significant hydrolysis, AN-125, a 20–30% AMPS copolymer, proves to be a viable option. Another research was conducted by Gaillard et al.^38^, where they attempted to enhance the chemistry of NVP copolymers by incorporating ATBS in the chain. It was found that chemical modifications to the polymer structure, incorporating functional groups like acrylamide (AM), sodium acrylate (AA), sodium acrylamido tertiobutyl sulfonate (ATBS), and N-vinylpyrrolidone (NVP), have yielded significant improvements in long-term thermal and chemical stability. Diverse polymer prototypes were formulated for the study through adjustments in the composition of these functional groups. Employing NMR structural analysis across various conversion rates, enhancements have been made to the bulk gel polymerization process, an industrial-scale method for producing EOR polymers. The most promising polymer candidates underwent rigorous stability testing. The top-performing polymer prototype's thermal stability was assessed under conditions of residual oxygen ranging from 0 to 200 ppb, at temperatures up to 120 °C, and in saline environments with salinities reaching 70 g/L TDS, featuring high water hardness. Furthermore, one-year aging experiments were conducted at 105 °C, 120 °C, and 140 °C under anaerobic conditions and with an oxygen concentration of 200 ppb. The results demonstrated marked improvements in the thermal and salinity stability of the polymer structure when NVP and ATBS were present together in specific amounts. It was found that ATBS might considerably improve the stability of NVP terpolymers by replacing acrylic acid in the polymer chain. Some of the meticulously engineered polymers, with chemical structures optimized for maximum stability, displayed impressive resilience in harsh reservoir conditions, exhibiting minimal viscosity loss even after a year of aging. In coreflooding experiments, these polymers showcased excellent propagation characteristics in carbonate rock with permeabilities ranging from 100 to 150 mD. As a result, SAV 225 (containing 20–30 mol% NVP) and SAV 333 (containing 30–45 mol% NVP) emerged as the most effective polymers at temperatures of 105 and 120 °C, respectively. Consequently, this study conducted by Gaillard et al.^38^ underscores the effectiveness of polymer flooding in reservoirs facing challenging conditions characterized by high salinity and elevated temperatures. The thermally resistant polymers developed through this research exhibit promise not only for alkaline surfactant polymer systems but also for surfactant polymer floods, along with other applications within the oil and gas industry demanding stability under high-temperature and high-salinity conditions. Incorporating these innovative polymers helps maintain a favorable mobility ratio during their transit from injection wells to production wells, ensuring consistent polymer viscosity as it propagates through the reservoir.

Similarly, Dupuis et al.^33^ performed screening studies utilizing polymers with varying amounts of ATBS, while one polymer was composed of both NVP and ATBS monomers. These were a new class of highly thermally stable synthetic polymers, and their propagation behavior was studied in this work. These new synthetic polymers exhibited excellent thermal stability up to temperatures of 140 °C, typical in the harsh Middle Eastern brines, where salinity ranges from seawater to 220 g/L TDS. Through coreflood experiments with carbonate cores and Clashach sandstone cores with permeabilities ranging between 100 and 700 mD, the adsorption and mobility reduction of the NVP- and ATBS-composed polymers were assessed. Results showed reductions in permeability and mobility for the polymer, which proved effective propagation in both rock types. During rheological tests at a salinity of 100,000 ppm and a temperature of 25 °C, the observations indicated that due to the lower molecular weight of NVP, the required concentration for the polymer containing NVP was substantially higher than that of other polymers. Moreover, depending on the permeability of the reservoir, molecular weights can be tailored from low to high. However, the NVP-based polymers were deemed less desirable due to their higher price. In addition, for NVP-free polymers, the lowest concentration required to achieve the optimum polymer viscosity increased as ATBS content increased. It was believed that this was due to the tendency of ATBS to reduce the molecular weight of the polymer, thereby decreasing the viscosity of the solution. In summary, the development of these innovative polymers signifies a substantial step forward in broadening the application of polymer EOR techniques within demanding reservoir conditions. Furthermore, a more comprehensive assessment through experimental investigations may be required to gain precise insights into aspects such as adsorption in carbonates and sandstones, tolerance to high salinity, and resistance to mechanical degradation.

Furthermore, Alfazazi et al.^53^ evaluated three new NVP-HPAM-based polymers (SAV10, SAV28, and SAV37) to identify the best candidate for the harsh conditions existing in Middle Eastern reservoirs by subjecting them to a polymer screening study. In order to determine the efficacy of these novel polymers, a thorough rheological study was first carried out at various polymer concentrations (1000–4000 ppm) and brine salinities. Subsequently, a three-month thermal stability test was run in anaerobic conditions at 120 °C. In the end, Alfazazi and co-authors^53^ conducted an injectivity test using the best polymer at 120 °C and 167,000 ppm formation salinity without the presence of oil. Three different polymer concentrations were sequentially injected into the experiment including 3000, 1500, and 750 ppm. During the course of this study, variables such as resistance factor, residual resistance factor (RRF), in-situ rheology, and apparent shear rates were examined. One hundred days of aging revealed that SAV10, with a molecular weight of 2–4 million Daltons, sustained 90% of its viscosity at a temperature of 120 °C and a salinity of 167,000 ppm. All three polymers exhibited satisfactory initial viscosifying characteristics at room temperature and demonstrated shear-thinning behavior within the shear rate range of 1–100 s^−1^, as indicated by the rheometric study results. The observations further revealed that polymer viscosities declined with rising temperature and salinity levels. However, they maintained considerable resistance even in conditions reaching up to salinity of 167,000 ppm and temperatures of 120 °C. The SAV10 polymer was more stable at increasing temperatures and maintained a greater viscosity than the other two polymers. This was due to the increased ATBS concentration in the SAV10 chain’s backbone. Moreover, during the injectivity test, dilatant behavior was seen at high flow rates, while shear-thinning was observed at lower injection velocities, supporting the complex flow behavior of SAV10 in porous media. The resistance factor (RF) of the novel SAV10 polymer ranged from 20 to 10 when injecting at 3000 ppm (at various flowrates). The RF ranged between 14 and 6.5 as well as 5 and 2.7 at 1500 and 750 ppm, respectively. Shear-thinning behavior was seen at low polymer injection flowrates (0.05–1.0 cc/min). On the other hand, shear-thickening behavior was seen at all concentrations at high flowrates. Ultimately, it was discovered that the residual resistance factor (RRF) for the injectivity experiment was 6.17. Therefore, for potential application in a heterogeneous carbonate reservoir with a higher salinity and temperature of 120 °C, the novel SAV10 polymer demonstrated favorable findings.

Hence, as evinced by the above laboratory discoveries, the monomers ATBS and NVP can promote acrylamide robustness and stability in carbonate reservoirs. Although these new polymers may cost 3–10 times more than traditional HPAM polymers, they are recommended to be used if the project makes a profit^38^. However, more studies are required to understand and control the viscoelasticity of these polymers, further increasing oil recovery and improving project economics.

#### Application of polymer nanohybrids under harsh conditions

A great deal of emphasis has been given to the use of polymer solutions in enhanced oil recovery (EOR) methods because of their role in improving sweep efficiency during the oil recovery process^54–56^. Similarly, nanoparticles have drawn a lot of attention for enhancing oil recovery (EOR) owing to their distinctive features^57,58^. Despite extensive research being done in these domains (i.e., polymers and nanoparticles), the impact of nanoparticles on the effectiveness of polymers under harsh conditions (i.e., high-temperature and high-salinity, HTHS) has not been fully studied. We present several studies analyzing the effect of nanoparticles on polymer flooding efficiency below.

Maghzi and co-authors^59^ employed polyacrylamide solutions and DSNP at varying salinity levels to scrutinize the impact of silica nanoparticles on the performance of polyacrylamide in the presence of salts during heavy oil polymer flooding. In their research work, a specialized quarter-five-spot glass micromodel, saturated with heavy oil, served as the experimental platform for a comprehensive series of polymer flooding experiments. The study also involved conducting viscosity measurements to facilitate a thorough analysis of the results obtained from the polymer flooding tests. Additionally, precise microscopic monitoring techniques were employed to evaluate the distribution of residual heavy oil and the polymer solution at the pore level. The findings indicate that as salinity increases up to the threshold value of 24,000 ppm, the oil recovery during flooding with 1000 ppm polymer and nanosuspension decreases. However, beyond this salinity level, there is a slight increase in the oil recoveries for both polymer flood and nanosuspension flood. Moreover, at the same salinity level, the oil recovery for nanosuspension flooding is approximately 10% higher than that for polymer flooding when silica nanoparticles are absent in the polymer solution. Furthermore, microscopic images reveal an enhancement in displacement efficiency with an increase in nanoparticle concentration. This enhancement can be attributed to the capability of silica nanoparticles to prevent polymer degradation in the presence of salts. Within the same salinity range for both the polyacrylamide solution and the silica nanosuspension, the viscosity of the nanosuspension exceeds that of the polymer solution. In essence, when nanoparticles are introduced into the polyacrylamide solution, they are in competition with the polymer molecules for attracting the cations. As a result, the ion–dipole interaction between the nanoparticles and cations diminishes the degradation of the polymer molecules.

In another study, Maghzi et al.^60^ investigated the impact of nanoparticles on rock wettability and its subsequent influence on oil recovery in polymer-based EOR processes. They conducted several injection experiments using a five-spot glass micromodel that had been saturated with heavy oil. The injection fluids included a polyacrylamide solution and a solution of dispersed silica nanoparticles in polyacrylamide (DSNP). The experiments revealed changes in the contact angles of the glass surface at various wettability levels after it had been coated with heavy oil. The results demonstrated that the inclusion of silica nanoparticles led to a 10% increase in oil recovery during polymer flooding. Furthermore, the flooding tests in the pores and throats revealed a significant shift toward water-wetting of the medium due to the presence of the DSNP solution. Sessile drop experiments also showed that coating a surface with heavy oil could render it oil-wet. However, the wettability of a surface could be partially altered to become water-wet by coating it with distilled water and a polymer solution, while a strongly water-wet surface could be achieved by coating it with DSNP.

Similarly, Daneshmand and colleagues^61^ corroborated the favorable impact of nanoparticles on altering rock-wettability, specifically transitioning an oil-wet condition of the rock to a water-wet state, as part of the enhanced oil recovery (EOR) process. Their research outcomes revealed that the incorporation of trimethoxy (propyl) silane, acting as a hydrophobic agent, onto the surface of modified silica nanoparticles within polyethylene glycol methyl ether notably enhanced retention and modified wettability. This effect was particularly pronounced on oil-wet substrates influenced by hydrophobic interactions. Under conditions of 1000 ppm concentration and within a salinity range of 2000–40,000 ppm, the modified silica nanoparticles, in conjunction with mixed polyethylene glycol methyl ether (Mn5000) and trimethoxy (propyl) silane, exhibited promising performance.

Furthermore, Giraldo and co-authors^62^ delved into the impact of SiO_2_ nanoparticles on the thermal stability and rheological properties of polymeric solutions based on hydrolyzed polyacrylamide (HPAM). They prepared nanofluids by mixing HPAM with a fixed concentration of nanoparticles in an aqueous solution. The authors conducted batch-mode experiments to establish HPAM adsorption isotherms over the SiO_2_ nanoparticles. These isotherms were then modeled using the Langmuir, Freundlich, and Solid–Liquid Equilibrium (SLE) models. The SLE model provided the best fitting when evaluated by the root-mean-square error (RMSE %). The results of the adsorption experiment, specifically the adsorption of polymer onto the nanoparticles, indicated Type III behavior in the isotherms. Additionally, it was observed that there was minimal polymer desorption from the nanoparticle surfaces, suggesting that under the conditions studied, the sorption process could be deemed irreversible. In the rheological tests conducted across a temperature range of 25 to 70℃ for all SiO_2_-HPAM combinations examined, a consistent non-Newtonian behavior was observed.

Finally, Haruna et al.^63^ utilized graphene oxide (GO) nanosheets to enhance the resilience of HPAM at high temperatures. The dynamic and viscoelastic behavior of stable GO dispersions in aqueous HPAM was investigated. It was revealed that the addition of GO substantially improved the base polymer fluid viscosities, high-temperature stability, and elastic properties of a dispersion. According to the observed spectral data, the GO and HPAM functional groups formed covalent bonds and electrostatic hydrogen bonds, which improved stability and viscosity suitable for oil recovery at high temperatures.

## Polymer viscoelasticity

Viscoelastic materials possess the unique quality of displaying both viscous and elastic attributes when subjected to deformation^14,64^. To grasp the concept of fluid viscoelasticity, it is helpful to begin with the well-established behaviors of viscous fluids and solid materials with elastic properties. In the case of a simple viscous fluid, its behavior adheres to Newton's law of viscosity as follows^65^:

1 $$\tau =\mu \dot{\gamma } ,$$

where $\tau$ is the shear stress, $\dot{\gamma }$ is the shear rate, and $\mu$ is the viscosity. Note that $\mu$ is constant for a Newtonian fluid, while it is a function of shear rate for pseudoplastic or dilatant fluids.

Conversely, elastic substances tend to revert to their original configurations when subjected to minor deformations. In the context of an ideal solid subjected to shear stress, Hooke's law holds true for small displacements^14^:

2 $$\tau ={G}^{\prime}\gamma ,$$

where ${G}^{\prime}$ is the elastic modulus and $\gamma$ is the strain.

It is worth noting that the basic Hooke's law behavior of stress in a solid is equivalent to Newton's law for fluid stress. The shear stress in a simple Newtonian fluid is related to the rate of strain or shear rate, but it is proportional to the strain itself in a Hookian solid. When dealing with a viscoelastic fluid that displays a combination of viscous and elastic characteristics, the equation governing shear stress needs to encompass both Newton's and Hooke's principles. The Maxwell model shown below offers a potential constitutive relationship between stress and strain for such a fluid. This model assumes a purely viscous damper, as defined in Eq. (1), and a purely elastic spring, as outlined in Eq. (2), that are interconnected in a series configuration^14^:

3 $$\frac{\tau }{\mu }+\frac{1}{{G}^{\prime}}\left(\frac{\partial \tau }{\partial t}\right)=\dot{\gamma } ,$$

This equation has the proper limiting behavior: it converges to an equation applicable to a standard Newtonian fluid in cases where the rate of change of shear stress $\left(\partial \tau /\partial t\right)$ approaches zero, indicating steady shear flow. On the other hand, when the stress experiences rapid variations with time, and $\tau$ becomes insignificant relative to $\left(\partial \tau /\partial t\right)$, the equation transforms into the constitutive equation characterizing a Hookeian solid. Different versions of the Maxwell model have been proposed in the literature and can be found elsewhere^66^. Additionally, models such as Oldroyd-B and Finitely Extensible Nonlinear Elastic-Peterlin (FENE-P) prove instrumental in characterizing the flow of viscoelastic polymers in porous media. The Oldroyd-B model is a linear viscoelastic model that incorporates the polymer ability to store and release energy in response to deformation. The FENE-P is a more sophisticated model that takes into account the finite extensibility of polymer chains and provides a more accurate description of viscoelastic behavior. It is encouraged that readers refer to the work by Zhang et al.^67^ for a thorough understanding of these models.

As we explore viscoelastic materials further, we may find that their unique characteristics have practical applications, especially in EOR. Polymers like hydrolyzed polyacrylamides, with their flexible structures, exhibit notable viscoelastic properties within porous media under favorable conditions. This distinctive behavior holds significant potential for improving oil recovery rates. In the context of viscoelastic polymer flooding in porous media, two prevalent flow regimes come into play: shear-dominant and extensional-dominant. In shear-dominant flow, polymers often exhibit pseudo-plastic or shear-thinning behavior, meaning that their apparent viscosity decreases with increasing shear rate. This behavior can help reduce frictional losses within the reservoir, thus promoting better fluid injectivity and displacement^68^. On the other hand, in the extensional-dominant regime, viscoelastic polymers tend to display dilatant or shear-thickening behavior^69^. This means that their apparent viscosity increases dramatically under extensional conditions^70^. This behavior is especially advantageous in porous media, where it contributes to enhanced sweep efficiency by creating higher resistance to flow in narrow pore throats, leading to a more uniform oil displacement.

It is essential to emphasize that this unique dilatant behavior of viscoelastic polymers is a phenomenon primarily observed in porous media and is not typically encountered in weak bulk shear fields^16,71^. This observation underscores the pivotal role that the porous structure of the reservoir plays in altering the rheological response of viscoelastic polymers. The confinement and tortuosity of pore spaces in porous media significantly influence polymer chain conformation, resulting in this distinctive shear-thickening behavior. One possible explanation for this phenomenon lies in the alternating expansion and compression experienced by viscoelastic polymers as they navigate the converging and diverging flows within porous media—transitioning from pore bodies to throats^29,72^. The deformation frequency rises with flow velocities and prevents the polymer from swiftly returning to its original shape after being disturbed. Consequently, this deformation results in an elastic strain, causing an increase in pressure drop and the emergence of shear-thickening behavior^73,74^.

Viscoelastic polymers, with their dual viscous-elastic nature, offer a promising avenue for enhanced oil recovery in porous media. Understanding the complex interplay between their rheological behavior and the porous medium is essential for optimizing their application and realizing their potential benefits. In the following sections, we attempt to provide thorough insights into viscoelastic polymer flooding, discussing primary parameters used for polymer viscoelasticity, describing various models to characterize polymer rheological behavior in porous media, and explaining the factors that can affect and control polymer viscoelasticity.

### Deborah number

Deborah number ($De$), the ratio of elastic to viscous forces, is one of the most important variables for measuring the flow of viscoelastic polymers in porous media^75^. $De$ may be characterized as follows^76^:

4 $$De=\frac{{\tau }_{r}}{t} ,$$

where $t$ and ${\tau }_{r}$ are residence and relaxation times, correspondingly. The small Deborah numbers result in fluid-like behavior, whereas a material with large $De$ would have solid-like behavior^77^. Therefore, as per the equation above, the solid-like response is expected in the case of high elastic material (significant relaxation time) or a quick deformation process (short residence time)^78^.

It was found that the viscoelastic impact is insignificant for small Deborah numbers because the fluid instantly responds to its local deformation state where it does not "remember" its initial configurations. Furthermore, upon reaching the critical $De$, viscoelastic behavior becomes more pronounced and is accompanied by a considerable effect on pressure drop and elongational viscosity^79^. However, there is no agreement among the researchers on a particular critical Deborah number, and the proposed values substantially differ in magnitude from 0.05 to 10 and even more^12,64,70,72,79,80^. The large variations in suggested threshold Deborah numbers are primarily attributed to the inconsistent residence time calculations. Later in this work, we will explore the various approaches to determine residence time.

Furthermore, it was reported that the Deborah number determined by oscillatory relaxation time is inadequate for analyzing the impact of viscoelastic polymer flooding on oil recovery^12,81–83^. Garrouch and Gharbi^83^ showed comparable oscillatory relaxation times for HPAM and Xanthan polymers, contrary to the broadly accepted opinion that synthetic polymers, being more flexible and elastic, would have considerably longer relaxation times in the porous medium. They raised concerns about using the $De$ derived from the oscillatory relaxation time and suggested a new factor called viscoelasticity number (${N}_{v}$), as described below^83^:

5 $${N}_{v}=\frac{\sqrt{\phi k}}{{\tau }_{r}{{u}_{w}}^{\overline{n }-1}} ,$$

where $k$ is the absolute permeability,$\phi$ is the porosity, ${u}_{w}$ is Darcy’s velocity, and $\overline{n }$ is the mean power-law constant inside the porous medium. Even though the authors stated that their term could distinguish between viscous and viscoelastic flow and consider polymer retention in porous media through $\overline{n }$, the viscoelasticity number has not been used as often as the Deborah number in recent studies. Therefore, further investigations may be required to utilize ${N}_{v}$ and justify the proposed model’s advantages over $De$.

#### Relaxation time

**Figure 3:**
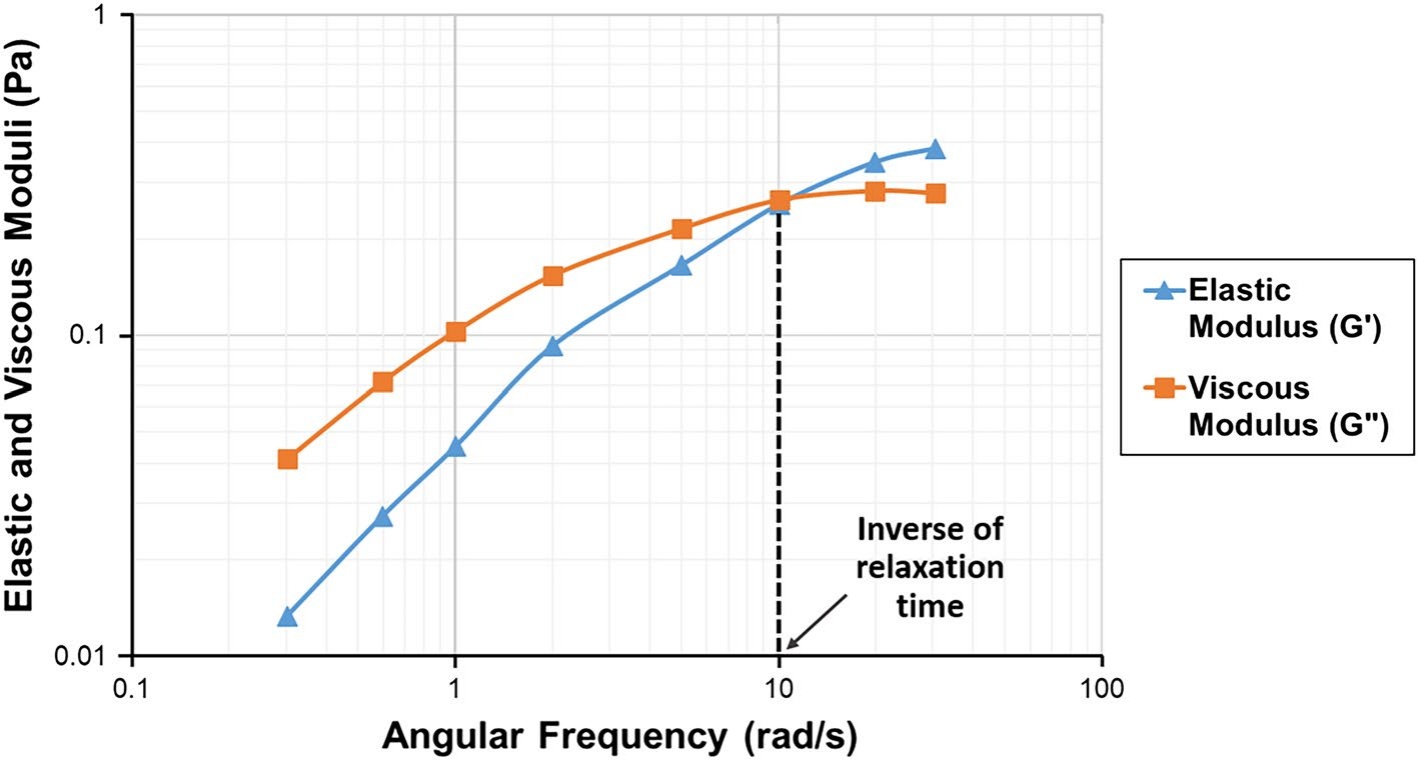
The Cross-Over Point Model to determine relaxation time.

Relaxation time is the time required for the polymer to return to its initial shape after being deformed during the diverging and converging flows in porous media. It is expected that the viscoelastic properties of a polymer solution will be more apparent if the solution undergoes a rapid deformation where the residence time is close to its relaxation time. Several models might be used to estimate polymer oscillatory relaxation time, including "The Rouse model"^84^ and "The G’ and G” Cross-Over Point Model"^85^. The Rouse model can determine relaxation time by fitting it to the experimental data. This model introduces the polymer molecule as a flexible chain of N beads connected by N-1 elastic springs. Moreover, a molecule has N-1 relaxation times associated with the viscous and elastic moduli of the model when immersed in a solvent fluid^72^. Since critical Deborah numbers are first obtained for the longest relaxation time, the most extended spectrum of the obtained relaxation times is used for the calculations and injectivity tests. On the other hand, according to the Cross-Over Point model, the oscillatory relaxation time of a polymer solution is equal to the inverse of the angular frequency at which the viscous (G") and elastic (G′) moduli intersect (Fig. 3). Despite its simplicity, the Cross-Over Point Model estimates oscillatory relaxation time accurately^18,86^. However, it is recommended to use this technique for highly viscoelastic polymer solutions^22^.

In addition, several empirical correlations have also been presented to predict the polymer relaxation time. One of them, developed by Kim et al.^28^, is based on the generalized Maxwell model and is presented below:

6 $${\tau }_{r}={A}_{1}{{C}_{p}}^{2}+{A}_{2}{C}_{p}+{\tau }_{0} ,$$

where ${\tau }_{0}$, ${A}_{1}$, and ${A}_{2}$ are the empirical constants and ${C}_{p}$ is the polymer concentration. Secondary correlations to compute the empirical parameters of the model were developed based on experimental data and can be found elsewhere^28^. Although the method can determine relaxation time using brine hardness, salinity, and polymer concentration, the challenges related to the complexity of empirical equations and the number of fitting parameters question the efficiency and practicality of the described model.

**Figure 4:**
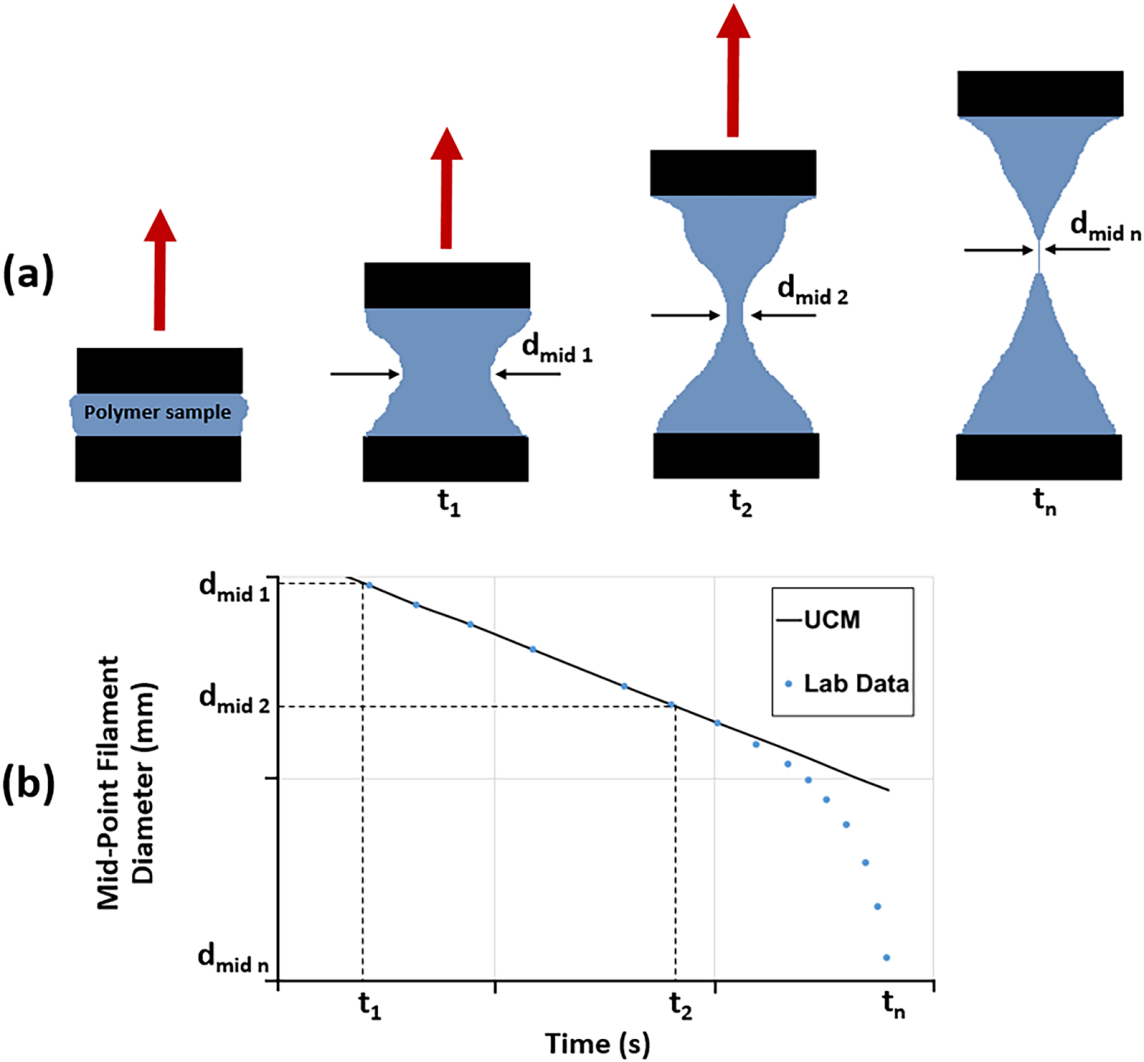
Measurements using CaBER: (**a**) polymer placed between two plates changes in filament diameter as one plate is rapidly detached from another, (**b**) the laboratory data are fitted using the Upper-Convected Maxwell model (UCM) to estimate extensional relaxation time by regression (modified after Azad and Trivedi^81^).

In contradiction to the above, it is recently reported that using extensional relaxation time rather than oscillatory relaxation time could lead to more reliable results since the former is more representative of a porous medium^12^. The extensional relaxation time is determined in bulk extensional, shear-free flow and is often higher than the oscillatory relaxation time. Because of the large normal stresses induced in pore constrictions, there is strong extensional flow in porous media. The substantial extensional stresses cause considerable deformation of the polymer as it flows through the pore throat area. On the other hand, testing the polymers in a weak bulk oscillatory field results in quite limited deformation, which cannot account for their behavior in the porous medium^21^. Consequently, future research may focus on measuring extensional relaxation times to produce more accurate results. In this regard, the filament stretching extensional rheometer (FiSER)^87,88^ and the capillary breakup extensional rheometer (CaBER)^12,89^ are commonly applied approaches for measuring the elongational relaxation time. In the former technique, the end plates are separated exponentially, resulting in a constant deformation rate^22^. On the other hand, in the CaBER applications, the polymer solution is placed between plates, and the variation in filament diameter is measured while the top plate is rapidly detached from the lower plate. Subsequently, the acquired filament diameter-time measurements are fitted using the upper-convected Maxwell model in a semi-log plot, and the extensional relaxation time is determined through the regression^81^. The latter technique is illustrated in Fig. 4. The various approaches for estimating polymer relaxation time are more thoroughly discussed elsewhere^22,90,91^.

In summary, the relaxation time can be measured experimentally or computed using empirical correlations. As noted above, extensional relaxation time might lead to more accurate Deborah number predictions and oil recovery projections by being more representative of the porous medium than the oscillatory measurements. However, it is essential to note that the extensional relaxation time is derived from pure elongation in the extensional bulk field, whereas in the porous media, the combination of shear and elongation exists. Therefore, applying a downscaling factor to extensional measurements is recommended to capture the shear field effects. However, determining the accurate downscaling factor is still challenging and requires further clarification.

#### Residence Time

Residence time refers to the duration of a polymer flow in porous media from one constriction to another^92^. In Deborah number computations, the residence time is often used as the inverse of the velocity gradient, either strain rate ($\dot{\varepsilon }$) or shear rate ($\dot{\gamma }$). Strain rate ($\dot{\varepsilon }$) (stretch or elongational rate) has been used to calculate the resident time by several researchers^12,72,80,93^. The most frequently used expression for the strain rate is given below^93^:

7 $$\frac{1}{t}=\dot{\varepsilon }=\left[\frac{{u}_{w}}{\left(1-\phi {S}_{w}\right)\sqrt{150{k}_{w}/{S}_{w}\phi }}\right] ,$$

where ${S}_{w}$ and ${k}_{w}$ are the aqueous phase saturation and effective permeability, respectively.

On the other hand, some authors used the shear rate ($\dot{\gamma }$) to determine the residence time^73,94^. The shear rate is usually expressed by the following equation^95^:

8 $$\frac{1}{t}=\dot{\gamma }=C\left[{\left(\frac{3{n}_{1}+1}{4{n}_{1}}\right)}^{{n}_{1/\left({n}_{1}-1\right)}}\left(\frac{4{u}_{w}}{\sqrt{8{S}_{w}\phi {k}_{w}}}\right)\right] ,$$

where $C$ is the shear correction factor and ${n}_{1}$ is the shear-thinning index. The value of $C$, representing the contrast between the actual porous media and the equivalent capillary tube, is determined by the transport parameters and petrophysical properties^64,96,97^.

Moreover, the Deborah numbers calculated using the shear rate will be larger than that determined by the strain rate. It is because of the smaller coefficient in the denominator of the shear rate^21^. The discrepancies between shear rate and strain rate implementations mainly arise while defining the onset of viscoelastic behavior^48,77,98–100^. This inconsistency in residence time estimation leads to various critical Deborah numbers reported in the literature. The issue regarding the viscoelastic onset will be discussed in the next section in more detail.

### Viscoelastic polymer flow in the porous medium

#### Flow regimes exhibited by viscoelastic polymers

**Figure 5:**
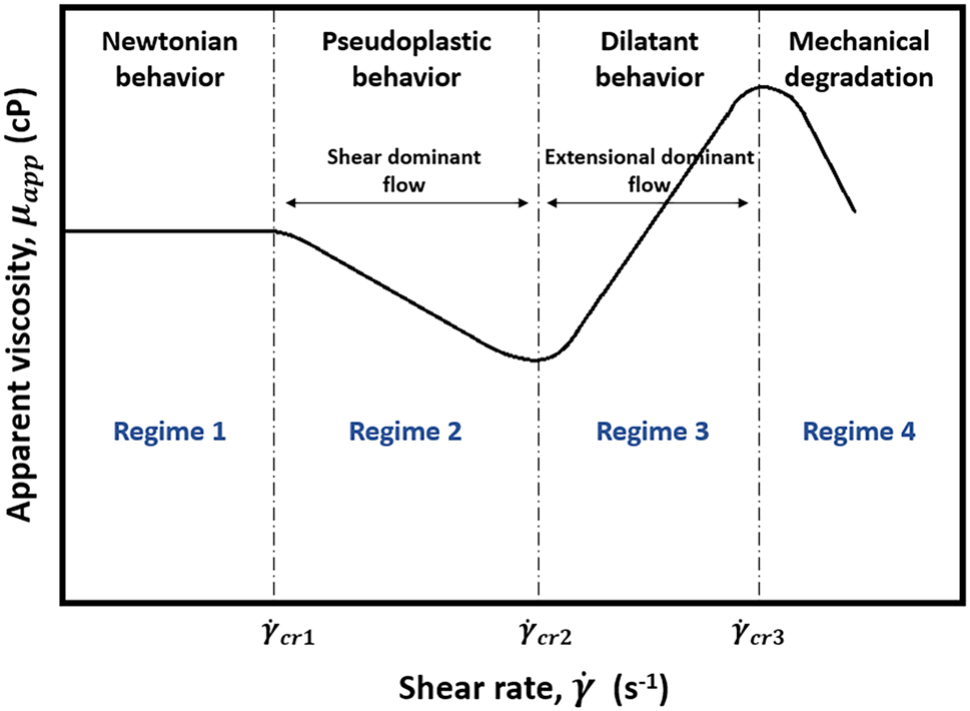
Schematic illustration of viscoelastic fluid flow behavior^113^.

Different flow regimes may be observed in porous media when viscoelastic polymers are involved (Fig. 5). During Regime 1, which occurs at low shear rates, the entropic forces are greater than the fluid flow-induced hydrodynamic drag forces. As a result, the polymers are maintained in the form of a coil and act as a Newtonian fluid with constant viscosity. However, as the shear rate continues to increase and reaches the first critical rate (${\dot{\gamma }}_{cr1}$), the polymer molecules are exposed to increased drag forces, which cause the polymer coils to unravel and realign themselves along the direction of flow, thereby reducing polymer viscosity^75,101^. In other words, in this shear-dominant Regime 2, polymers display shear-thinning or pseudoplastic behavior. However, under practical injection conditions, the degree of pseudoplasticity in porous media for most synthetic polymers is minimal or even nonexistent (${\dot{\gamma }}_{cr1}$=${\dot{\gamma }}_{cr2}$)^102–104^. For instance, in the case of the HPAM applications, the polymers usually exhibit only Newtonian and shear-thickening regimes in low and high shear rate regions, respectively. However, shear-thinning behavior might also be observed, but exclusively at low salinity or high polymer concentrations^101^.

Furthermore, viscoelastic polymers exhibit shear-thickening or dilatant behavior in the regions where shear rates exceed the second critical shear rate (${\dot{\gamma }}_{cr2}$) (Regime 3). It is important to note that the shear-thickening in Regime 3 is associated only with flexible synthetic polymers and is usually not observed while utilizing viscous biopolymers^16,104^. The dramatic increase of polymer viscosity in this regime is related to the so-called elastic strain. It is obtained by viscoelastic polymers at high shear rates when they flow through a sequence of pore throats and bodies in a strong extensional field^14^. The onset shear rate for shear-thickening, where viscoelastic effects are first pronounced, depends on various rock and fluid properties. For instance, ${\dot{\gamma }}_{cr2}$ decreases with increasing polymer molecular weight and concentration and decreasing solution salinity, temperature, and permeability of the rock^72,98^.

In some studies, the term extensional thickening or strain hardening (viscosity rises with strain) is preferred over shear-thickening since the increase in viscosity during extensional flow is obtained by extensional forces rather than shear^21,105^. In this case, the term shear-thickening is traditionally used to characterize the thickening that is encountered in bulk shear or mixed core-scale flows, while strain hardening refers to the thickening of the polymer solution in the bulk extensional field^21^. The importance of distinguishing shear-thickening and strain hardening emerged while defining the onset of thickening. It was revealed that polymer thickening begins at significantly lower fluxes in the extensional field than in the shear field or core-scale porous media, which was expected due to the shear field being much weaker than the extensional field^106^. In particular, a number of studies indicated that polymer strain hardening occurs at lower strain rates compared to shear-thickening in the shear field for the same polymers, which commenced at much greater shear rates^48,69,98–100^. It may justify additional oil recovery achieved by viscoelastic polymers, even at minimal fluxes, below the shear-thickening onset during core-scale flooding^16,64,107,108^. For example, Clarke et al.^16^ and Qi^64^ reported a decrease in residual oil saturation caused by viscoelastic synthetic polymers at low injection rates of around 1 ft/day, even though the shear-thickening onset had not yet been achieved. However, in order to accurately characterize viscoelastic polymer flow in porous media, where both shear and extensional stresses are present, it is essential to either establish a solid connection between these terms or stick to one of them to obtain consistent results.

Furthermore, after viscoelastic polymers experience complete stretching during shear-thickening flow, they build excessive stresses that eventually result in mechanical degradation (Regime 4). Degradation commences at ultimate shear rates higher than (${\dot{\gamma }}_{cr3}$), where maximum elongational viscosity starts to decrease because of the chain scission of polymer molecules. It is reported that injecting high molecular weight polymers into tight formations accelerates the degradation process, shifting ${\dot{\gamma }}_{cr3}$ to lower rates^108,109^. Mechanical degradation can indeed impose substantial cost constraints on polymer flooding applications, potentially resulting in project failure. Therefore, it is crucial to carefully consider and address this issue in order to optimize the efficiency and success of such endeavors. Mechanical degradation of viscoelastic polymers is comprehensively analyzed in Section "Mechanical degradation".

#### Viscoelastic models predicting polymer apparent viscosity

As discussed in the previous section, viscoelastic polymers might demonstrate different flow regimes in porous media. Therefore, various models are proposed to estimate the apparent viscosity of polymer and analyze its rheological response in porous media. These viscoelastic models (Table 2) are discussed below:

- *Unified viscoelastic model (UVM).* This model, provided by Delshad et al.^73^, calculates viscosity for both pseudoplastic and dilatant regimes. Based on this model, the apparent viscosity of a polymer is assumed to be equal to the summation of its shear and elastic viscosities, where shear viscosity is calculated through the well-known Carreau model^110^, and elastic viscosity is estimated by relating it to the relaxation time of a polymer. The viscoelastic onset and degree of shear-thickening in the UVM model can be adjusted using relaxation time (${\tau }_{r}$) and shear-thickening index (${n}_{2}$), correspondingly. Although bulk rheology tests can determine the model shear parameters (${\mu }_{\infty }$,${\mu }_{0}$, ${\lambda }_{1}$, ${n}_{1}$), time-consuming coreflooding measurements are still required to match model extensional parameters (${n}_{2}$, ${\mu }_{max}$). Nevertheless, the UVM overcomes the drawbacks of the previous models regarding the infinite rise in viscosity with the Deborah Number by utilizing ${\mu }_{max}$. However, this model does not accommodate mechanical degradation at high shear rates.
- *Extended viscoelastic model* Unlike the previously mentioned UVM model, the Extended Viscoelastic Model by Stavland et al.^111^ can capture the mechanical degradation of viscoelastic polymers. However, detailed coreflooding data are still necessary to accurately predict polymer shear-thickening and mechanical degradation. In this model, shear parameters are obtained by shear rheology experiments, while elongation and mechanical degradation parameters are determined from coreflooding tests and effluent sample analysis. Their model is represented by the sum of the shear and elongational viscosities multiplied by the mechanical degradation factor.
- *Azad-Trivedi viscoelastic model (AT-VEM)* proposed by Azad and Trivedi^81^ is based on the UVM. However, the authors eliminated the need for coreflooding experiments by using a capillary breakup extensional rheometer (CaBER) to measure model extensional fitting parameters (${\mu }_{max}$, ${\tau }_{ext}$, ${n}_{2}$). In this model, the downscaling factor of 0.35 was applied to ${\mu }_{max}$, while ${n}_{2}$ was used with the subtrahend of 1.2 to reduce the magnitude of these extensional parameters from pure elongation to the combination of shear and elongation usually faced in the porous medium. Similar to the original model, AT-VEM cannot accommodate the mechanical degradation encountered at ultimate shear rates.
- *Extended unified viscoelastic model (E-UVM)*. The Extended Unified Viscoelastic Model (E-UVM), provided by Zeynalli et al.^112^, is the extension of the Unified Viscoelastic Model (UVM), as the name indicates. In addition to the regimes covered by UVM, E-UVM can account for the viscosity reduction that happens at ultimate shear rates as a result of mechanical degradation. The mechanical degradation term of E-UVM is derived empirically using data gathered from coreflooding studies found in the literature. This concept introduces two key parameters: ${\lambda }_{3}$, which governs when mechanical degradation begins, and ${n}_{3}$, which regulates the severity of this degradation. In addition, the authors developed empirical correlations by using the machine learning approach to determine the model’s extensional and degradation parameters based on rock and polymer-solution characteristics^113^. Furthermore, the given model was tested in core-scale and field-scale simulation studies^114^. In general, the issue of mechanical deterioration of UVM was successfully resolved in E-UVM. However, further work is necessary to optimize the proposed empirical correlations for broader experimental conditions.

**Table 2:** Summary of viscoelastic models predicting polymer apparent viscosity.

No	Viscoelastic models	References
1	Unified Viscoelastic Model (UVM) ${\mu }_{app}={\mu }_{\infty }+\left({\mu }_{0}-{\mu }_{\infty }\right){\left[1+{\left({\lambda }_{1}\dot{\gamma }\right)}^{2}\right]}^{\left({n}_{1}-1\right)/2}+{\mu }_{max}\left[1-\mathit{exp}\left(-{\left({\lambda }_{2}{\tau }_{r}\dot{\gamma }\right)}^{{n}_{2}-1}\right)\right]$	Delshad et al.^73^
2	Extended viscoelastic model ${\mu }_{app}={\mu }_{\infty }+[\left({\mu }_{0}-{\mu }_{\infty }\right)(1+{{r}_{1}\dot{\gamma })}^{{m}_{1}}+({r}_{2}\dot{\gamma }{)}^{{m}_{2}} ]\cdot [1+{\left(d\dot{\gamma }{)}^{x} \right]}^\frac{j}{x}$	Stavland et al.^111^
3	Azad-Trivedi Viscoelastic Model (AT-VEM) ${\mu }_{app}={\mu }_{\infty }+\left({\mu }_{0}-{\mu }_{\infty }\right){\left[1+{\left({\lambda }_{1}\dot{\gamma }\right)}^{2}\right]}^{\left({n}_{1}-1\right)/2}+{\mu }_{max}^{0.35}\left[1-\mathit{exp}\left(-{\left({\lambda }_{2}{\tau }_{ext}\dot{\gamma }\right)}^{{n}_{2}-2.2}\right)\right]$	Azad and Trivedi^81^
4	Extended Unified Viscoelastic Model (E-UVM) ${\mu }_{app}=\left({\mu }_{\infty }+\left({\mu }_{0}-{\mu }_{\infty }\right)\cdot {\left(1+{\left({\lambda }_{1}\dot{\gamma }\right)}^{2}\right)}^{\frac{{n}_{1}-1}{2}}+{{\mu }_{max}[1-\mathrm{exp}(-(0.01{\tau }_{r}\dot{\gamma })}^{{n}_{2}-1})]\right){\left(1+{\left({\lambda }_{3}\dot{\gamma }\right)}^{2}\right)}^{\frac{{-n}_{3}}{2}}$	Zeynalli et al.^112^

Where ${\mu }_{0}$ and ${\mu }_{\infty }$ are the zero-shear rate and infinite-shear rate viscosities, respectively, ${\lambda }_{1}$ is the shear-thinning constant that represents a transition between Newtonian and pseudoplastic regions, ${\mu }_{max}$ is the maximum elongational viscosity, ${\lambda }_{2}$ is the shear-thickening constant that is usually taken as 0.01, ${n}_{2}$ is the shear-thickening (or strain hardening) index,${r}_{1}$ is the relaxation time constant, ${m}_{1}$ is the shear-thinning exponent,${r}_{2}$ is the inverse of elongation onset, ${m}_{2}$ is the elongational exponent, $d$ is the characteristic time constant for degradation, $x$ is a constant that is usually taken as 2, $j$ is the tuning parameter for degradation,${\tau }_{ext}$ is the extensional relaxation time,${\lambda }_{3}$ is the mechanical degradation constant, and ${n}_{3}$ is the mechanical degradation index.

**Figure 6:**
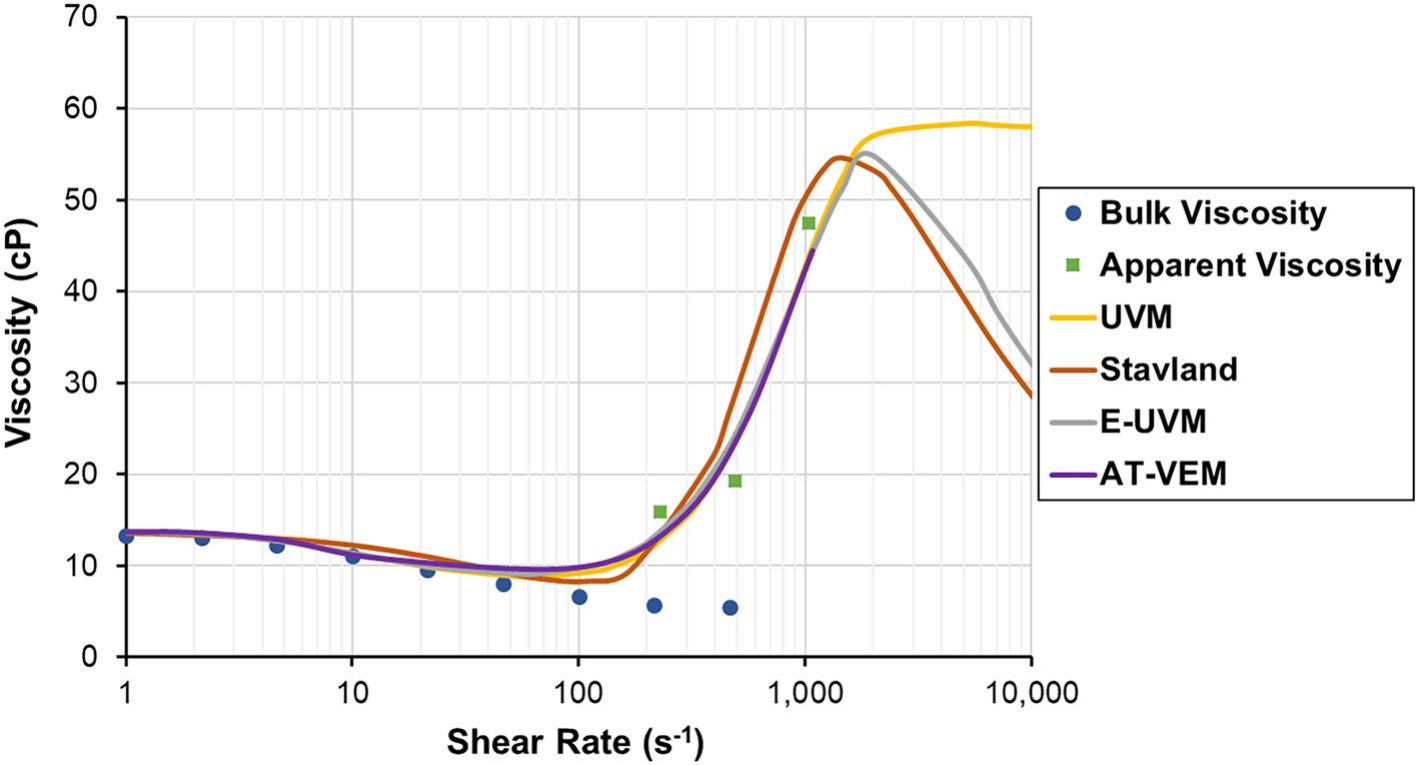
Comparison of viscoelastic models based on the experimental data from Magbagbeola^91^.

Figure 6 compares these models using the experimental data from Magbagbeola^91^. As stated earlier, E-UVM is the extended version of UVM, where mechanical degradation of viscoelastic polymers is accommodated. Moreover, compared to the model by Stavland et al.^111^, E-UVM was found to be more practical for fitting the laboratory data and adjusting the degrees and onsets of the dilatant and mechanical degradation regimes. The extensional fitting parameters utilized to fit the AT-VEM to the given lab data were obtained from Azad and Trivedi^81^.

#### Factors controlling viscoelasticity of polymers

**Figure 7:**
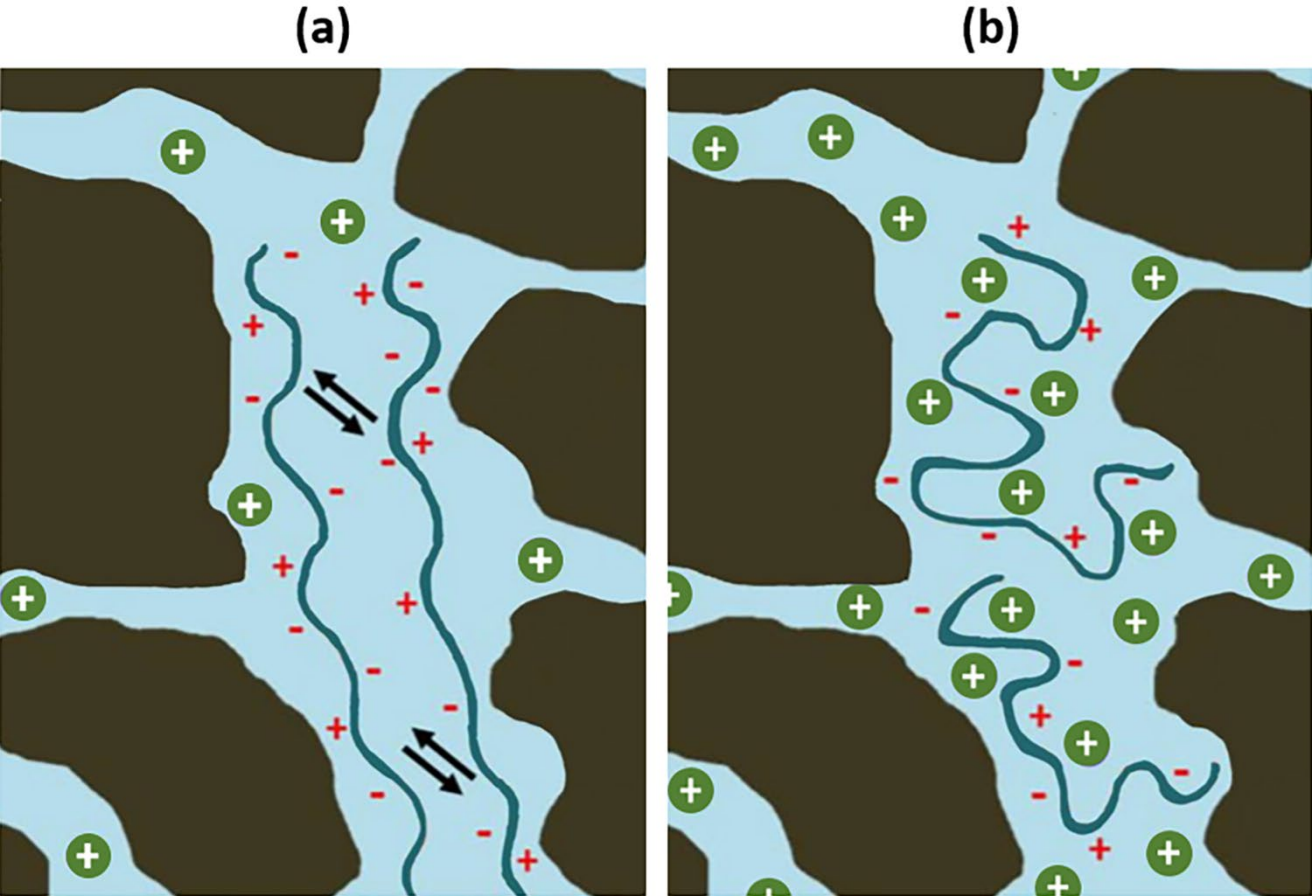
Schematic of charge-screening mechanism in porous media: (**a**) low-salinity condition, where polymers are stretched due to repulsive forces causing the higher flow resistance, (**b**) addition of salt cations to the brine creates a barrier for electrostatic interactions between the polymer chains, preventing polymer stretching and reducing its flow resistance. Herein, polymers are shown with blue curves, while cations are illustrated with solid green circles.
  However, the opposite results were obtained by Ait-Kadi et al.^105^. They investigated the in-situ behavior of Pusher-700 (HPAM) under various salinities and polymer concentrations. The authors revealed that the shear-thickening of dilute polymer solutions (170 ppm and 340 ppm) was enhanced with salinity. However, it is interesting to note that the effect of salts on polymer viscoelastic behavior was found to be negligible for semi-dilute solutions (510 ppm and 850 ppm). Apparently, the concept of the charge-screening mechanism cannot explain such results due to unusual intermolecular interactions existing at low polymer concentrations. Moreover, dilution provides more volume for salt counterions, scattering them at farther distances from macromolecules. As a result, the electrostatic charge shielding is reduced, and macromolecules expand, obtaining larger normal stresses and elastic viscosities. Additionally, increasing solution salinity contributes more counterions to such a dilute environment and enhances the flow resistance of the macromolecule. Furthermore, Sarsenbekuly et al.^126^ evaluated the hydrophobically modified PAM-based novel RH-4 functional polymer and compared it with conventional HPAM. The information about polymer properties and its molecular formula can be found elsewhere^126^. This work reported the positive salinity effect on the viscoelastic properties of the RH-4 polymer. The scanning electron microscope (SEM) tests showed that increasing solution salinity condenses the network structures of the novel polymer, enhancing its viscoelasticity. Additionally, another polymer, the so-called comb micro-block hydrophobic association polymer (CBHAP), was developed by Jiang and Pu^127^. Their polymer showed excellent shear-thickening at high salinities (>20,000 ppm). The rheological behavior of their polymer is illustrated in Fig. 8a^127^. As can be seen from the figure, the dilatancy was observed for salinities ≥ 20,000 ppm. Such results were related to the CBHAP structure and, particularly, the hydrophobic groups present in the polymer backbone. Here, changing polymer chains from curly to the stretched position at high shear rates played a significant role in enhancing polymer viscoelastic behavior under high salinity conditions.

The viscoelastic properties of the polymer may vary depending on the fluid flow conditions in the porous medium and polymer molecular characteristics. The factors controlling polymer viscoelasticity are discussed below:

- *Polymer concentration effect* According to a number of studies, polymer concentration is one of the factors improving polymer viscoelasticity^16,20,70,72,73,96,101,115–118^. Such a positive effect is related to more intensive interactions among the polymer molecules at higher concentrations. Under such circumstances, the polymer needs more time to return to its initial configuration after being deformed in the extensional field. As a result, polymer relaxation time is increased and, thus, improves polymer elasticity. However, some authors reported that the positive impact of polymer concentration disappears beyond a threshold concentration of 2500 ppm^119^.
- *Polymer molecular weight effect* The molecular weight of a polymer, like its concentration, also improves the viscoelastic response in porous media^16,73,91,119–121^. High M_w_ polymer with a considerable average length acquires a longer relaxation time and demonstrates substantial elasticity in porous media^119^. Moreover, it enhances the extent of polymer chain overlap and accelerates the elastic instabilities and dilatancy faced during viscoelastic polymer flow^22^. Although heavier polymers might exhibit quite strong viscoelastic behavior, there are also several disadvantages of using very high molecular weight polymers in field operations. Firstly, pore size distribution should be considered to avoid pore plugging and provide an adequate propagation of the injectant in the reservoir. Secondly, it is essential to note that the solubility of polymers is reduced with the increment in their molecular weight^22^. Finally, the mechanical degradation of polymers is also exacerbated with the M_w_ increase^109^. Therefore, rigorous planning and optimization of this factor are necessary before applying polymer flooding projects.
- *Temperature Effect.* Unlike the previous two factors, it is reported that temperature may significantly deteriorate polymer viscoelasticity^35,75,119,122,123^. In addition to the viscosity drop at elevated temperatures, it is also related to the thermal degradation of synthetic polymers; acrylamide moieties within HPAM hydrolyze to acrylate groups at temperatures above 70 °C, reducing polymer viscosifying and viscoelastic power, leading to its precipitation^33–35^. Moreover, the negative effect of temperature on polymer viscoelasticity can also be explained by the increased kinetic motions of individual atoms at higher temperatures. It might cause rapid structural rearrangements in the polymer chain and reduce its relaxation time^119^. Similar observations were also made by Alfazazi et al.^122^, where HPAM-based polymer was evaluated at various temperatures. It was found that although the polymer showed strong shear-thickening behavior at room temperatures, no thickening was observed at 90 °C.
- *Salinity effect* There is no consensus among the researchers on the effect of salinity on polymer viscoelasticity. Generally, it is widely accepted that the salt cations in saline solutions screen the negatively charged particles on the polymer backbone, reducing its viscosity^35,53^. In HPAM applications, for instance, the anionic charges of the carboxyl groups present in the HPAM backbone trigger intramolecular repulsions. As a result of these repulsive forces, the hydrodynamic radius of HPAM is enlarged, increasing the polymer viscosifying power. Therefore, the viscosity of the polymer solution can be considerably reduced at high salinities where external salt cations bond with carboxyl groups, preventing polymer stretching. According to several reports, this so-called charge-screening mechanism, illustrated in Fig. 7, can also reduce polymer relaxation time and deteriorate its elasticity^28,83,90,124,125^. In this regard, Garrouch and Gharbi^83^ measured similar oscillatory relaxation times for the flexible HPAM and rigid xanthan polymers in a high-salinity brine, while the former is far more elastic, with considerably higher relaxation time under normal conditions. Such a substantial drop in the oscillatory relaxation time of a synthetic polymer was related to its sensitivity to elevated salinities, which led to the collapse of the polymer molecular chain and much smaller random-coil structures.

**Figure 8:**
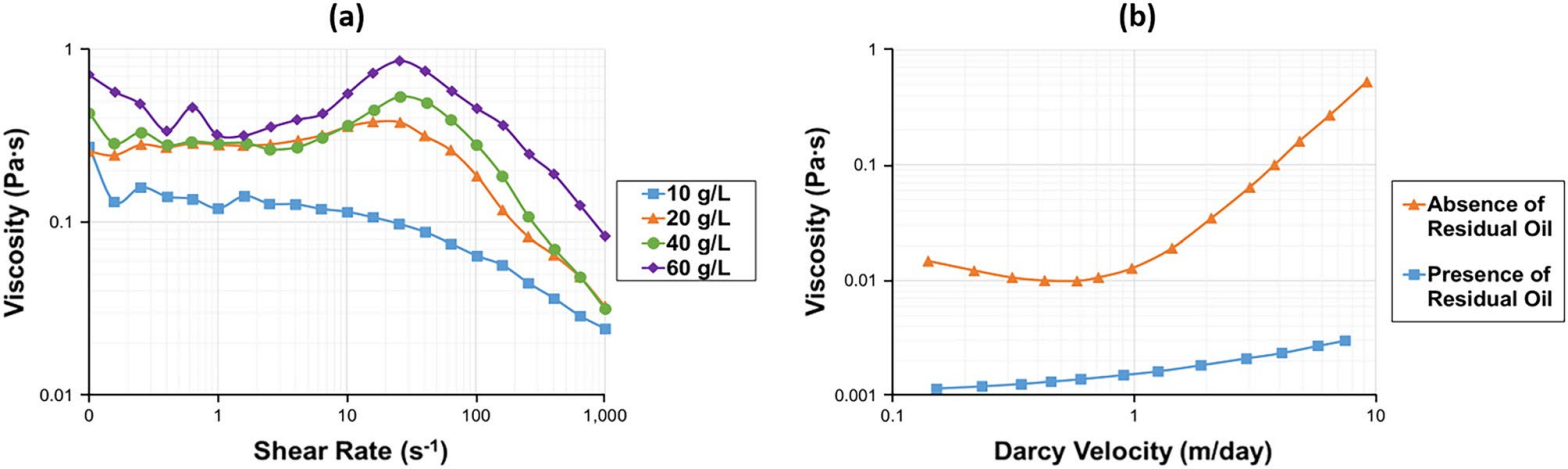
Rheology of polymers under various experimental conditions: (**a**) the effect of brine salinity on the viscoelasticity of CBHAP solution (C_p_ = 1.5 g/L; T = 45 °C) (modified after Jiang and Pu^127^), (**b**) the effect of residual oil saturation on the onset and degree of shear-thickening of Flopaam 3630S solution (C_p_ = 0.8 g/L; TDS = 4.7 g/L) (modified after Skauge et al.^75^).
  Azad and Trivedi^128^ also reported that salinity could actually enhance polymer elasticity and claimed that the oscillatory relaxation time decreasing with salinity is not able to properly represent the oil recovery by viscoelastic polymers at high salt concentrations. As a solution, they suggested measuring the extensional relaxation time. According to the authors, extensional relaxation time is directly proportional to brine salinity and may evaluate oil recoveries at various salinities more precisely than oscillatory relaxation time.

- *Water hardness effect* The effect of brine hardness on polymer elasticity also remains inconclusive. A group of scientists confirmed that high concentrations of divalent cations could considerably degrade the polymer and impair its elastic parameters^74,90^. In addition, several authors reported that the effect of hardness could be more pronounced than the comparative salinity effect. In particular, Koh^90^ noted that the detrimental impact of divalent calcium ions on FP 3630S relaxation time was 800 times stronger than that of monovalent sodium ions. On the contrary, Walter et al.^129^ claimed the positive effect of solution hardness. According to the authors, the divalent cations can bridge the anions on the polymer backbone, creating transient crosslinks among the polymer chains. Consequently, the elongated polymer at high hardness values would have a longer relaxation time and elastic viscosity.
- *Permeability effect* The reservoir permeability is another critical factor affecting the viscoelastic characteristics. Polymers flowing in tight reservoirs with low permeability exhibit greater viscoelasticity^72,93,130,131^. This might be due to a reduction in mean pore throat size, where higher differential pressures increase polymer deformation and trigger viscoelastic flow and the dilatant regime even at lower flow rates. However, it is essential to note that the onset of the mechanical degradation region is also shifted to lower rates in tight formations. Wang et al.^108^ recommended using high molecular weight polymers primarily in high-permeable rocks, while limiting polymer molecular weight in low-permeable reservoirs to minimize mechanical degradation issues. Choosing the right polymer for tight rock formations is crucial, as an unsuitable polymer can accelerate mechanical degradation, resulting in lower viscosity and viscoelasticity within the porous medium^132^. In this context, Ghosh and Mohanty^133^ have explored techniques like mechanical shear degradation and aggressive microfiltration to optimize HPAM polymer properties for successful injection into low-permeability carbonate reservoirs. Dynamic light scattering (DLS) measurements and comparison with pore throat distribution from mercury injection capillary pressure (MICP) have been used to determine the ideal shear degradation level. These innovative approaches have shown promise in enhancing the injection of polymer solutions into tight formations, underscoring the significance of pore throat distribution over permeability for effective polymer transport. However, further validation through additional coreflood experiments is necessary for full confirmation. Furthermore, according to Ghosh et al.^52^, when the polymer size exceeds the minimum pore dimensions, shearing becomes a valuable supplementary technique. For example, a 2000 ppm associative polymer solution, without shear degradation, could not flow through a 20-mD carbonate core. However, shear degradation reduced the particle size distribution and improved the transport of the associative polymer in low-permeability carbonate cores, allowing even a 5000 ppm AP solution to pass through a 26-mD core without causing blockages.
- *Effect of nanoparticles* It has been discovered that an alternative approach to enhance the viscoelastic properties of polymers involves the addition of nanoparticles to the polymer solution^63,134–136^. Haruna and colleagues^63^ conducted oscillatory tests on both pure HPAM (Hydrolyzed Polyacrylamide) and HPAM/GO (Graphene Oxide) composite systems. The outcomes obtained for the elastic modulus (G′) and viscous modulus (G") in relation to angular frequency (ω) reveal that the incorporation of GO into HPAM leads to a substantial improvement in the elastic properties. The samples exhibited reduced dependence on frequency in both G' and G", suggesting a more solid-like than liquid-like viscoelastic nature, especially when the concentration of GO is high. Moreover, the results indicate that, in comparison to pure HPAM, the composite solutions exhibit a longer relaxation time due to the presence of sufficient links and a network structure between the macromolecules. Similar findings were achieved in a study by Rahimi et al.^136^. They conducted experiments to examine the impact of sodium montmorillonite on oil recovery using a glass micromodel. The authors revealed that the interaction between montmorillonite and the higher molar mass polymer can function as a crosslinker, leading to the formation of a network structure. Rheological experiments involving high molar mass clay polymer nanocomposites (HCPN) and low molar mass clay polymer nanocomposites (LCPN) demonstrated that in HCPN, the elastic modulus exceeded the viscous modulus. This is attributed to the nanoclay acting as a crosslinker, thereby enhancing the strength of the viscoelastic network. Conversely, in LCPN, the viscous modulus surpassed the elastic modulus, indicating the absence of a formed network. As a result, the authors recommended the use of HCPN for field operations, as viscoelastic polymers exhibiting greater elasticity offer higher efficiency in oil displacement. Furthermore, Hu and co-authors^134^ developed innovative aqueous nanocomposites based on HPAM with SiO_2_ and explored their rheological characteristics across various salinity levels, temperatures, and aging times. The findings demonstrate that the introduction of silica nanoparticles (NPs) notably enhanced both the viscosity and viscoelastic properties of HPAM, particularly in high-temperature and high-salinity conditions. The Fourier transform infrared spectral data further validated that the enhanced performance resulted from the establishment of hydrogen bonds between the carbonyl groups in HPAM and the silanol functionalities on the surface of silica NPs. Moreover, oscillation testing revealed that the inclusion of SiO_2_ particles substantially promoted the crosslinks among polymer molecules, rendering the hybrids more elastically dominant. Finally, Khalilinezhad et al.^135^ conducted a study to investigate how adding dispersed Hydrophilic Silica-Nanoparticles (HSNPs) to a polymer solution affects oil recovery enhancement during polymer flooding. They conducted rheological studies to examine the impact of salinity, polymer concentration, and polymer molecular weight (M_w_) on the viscoelastic behavior of this combination. The measurements uncovered that the inclusion of HSNPs significantly affects the thickening characteristics of the polymer solutions, leading to enhanced viscoelasticity and salt tolerance in EOR polymers. However, it is important to note that the extent of this positive effect depends on the polymer concentration and its molecular weight. The beneficial influence of HSNPs was particularly evident in polymers with lower molecular weights.
- *Residual oil saturation effect* It was found that the residual oil saturation has a dual effect on polymer viscoelastic behavior. Generally, the in-situ rheology tests in the presence of oil have shown that polymers exhibit much weaker dilatancy; thus, have far better injectivity than the tests conducted at ${S}_{w}$=100%^40,75,103^. This can be one of the reasons why the actual injectivity experienced during viscoelastic polymer flooding operations is usually higher than the in-situ measurements in the absence of oil. However, it was also reported that the onset for shear-thickening is shifted to lower flow rates in the presence of oil^75,137^. It can be explained by narrower channels for polymer flow in the presence of immobilized oil on rock surfaces, inducing an earlier extensional flow regime.
  In summary, oil presence accelerates polymer shear-thickening and shifts its onset to lower shear rates. In fact, it might be detrimental from an injectivity perspective. However, residual oil saturation also has a reducing effect on pressure build-up and the degree of polymer shear-thickening^102^. Therefore, even though the polymer starts dilatancy earlier, its overall apparent viscosity is considerably lower in the presence of oil. An example of the in-situ behavior of viscoelastic polymer in the presence and absence of oil is illustrated in Fig. 8b^75^.

- *Hydrolysis effect* The viscoelastic characteristics of a polymer may also change depending on the extent to which the polymer is hydrolyzed^64,91,130,138^. It was found that the degree of hydrolysis increases the storage modulus and corresponding relaxation time, but its positive effect vanishes beyond a threshold value^130^. Ranjbar et al.^130^ observed the maximum elasticity for HPAM polymers at the hydrolysis level of around 27%. Additionally, the study by Qi^64^ revealed the enhanced benefits of hydrolysis on polymer viscoelasticity under specific conditions and polymer characteristics. Notably, hydrolysis can roughly double the relaxation time of the HPAM 3630 s polymer. Significantly, the impact of hydrolysis becomes even more pronounced, with relaxation time increasing by over two folds when the polymer concentration is elevated. Additionally, hydrolysis demonstrates increased effectiveness on polymers with substantial molecular weights, particularly those around 18 million Dalton. It is worth noting that the increase in relaxation time outweighs the increase in viscosity, especially at the apparent shear rates (ranging from 20 to 30 s^−1^) studied during polymer floods in this investigation^64^. Furthermore, it is important to remember that an increase in the degree of hydrolysis may lead to polymer precipitation at elevated brine hardness. Magbagbeola’s findings^91^ highlight that polymers with a higher degree of hydrolysis, particularly those exceeding 30%, exhibit increased responsiveness to divalent ions such as calcium and magnesium. This heightened sensitivity is observable in the behavior of HENGFLOC 63,020, which has a molecular weight of approximately 20 × 10^6^ Dalton and a degree of hydrolysis ranging from 28 to 30%. It is worth noting that, intriguingly, despite its higher molecular weight when compared to AN-125 (M_w_ of 8 × 10^6^ Dalton), which features a lower degree of hydrolysis, HENGFLOC 63,020 displayed lower viscous and elastic moduli across all frequencies tested.
- *Injection rate effect* The injection rate is a critical parameter that can significantly enhance the viscoelastic properties of polymers. When the injection rate is increased, the residence time of polymers within the system experiences a sharp reduction. This reduction, in turn, leads to a substantial increase in the corresponding Deborah numbers, as indicated by Eq. (4). Consequently, this heightened Deborah number enhances the elasticity of the polymer, resulting in improved viscoelastic effects^139^. In practical terms, this means that the most pronounced viscoelastic effects and shear-thickening behavior tend to manifest in the vicinity of wellbores where high flow rates are prevalent. However, it is important to emphasize that optimizing the injection rate requires a judicious approach. Several factors need to be considered, including the potential for mechanical degradation of the polymer, the risk of excessive formation fracturing, and other associated challenges. Careful attention must be paid to strike a balance between maximizing viscoelasticity benefits and mitigating potential drawbacks, ensuring the effective performance of polymer-based fluids in various industrial applications. Additionally, research has shown that the injection rate can also influence the dispersion of polymers within the fluid, impacting their effectiveness in diverse applications such as enhanced oil recovery, hydraulic fracturing, and drilling operations^140^. Therefore, a comprehensive understanding of the relationship between injection rate and polymer behavior is essential for optimizing these processes.
- *Pre-shearing effect* Shearing of polymers before injection can result in a significant variation in their viscoelastic properties. It is reported that the pre-shearing process shifts the onset of dilatancy to higher rates and reduces the degree of polymer shear-thickening^102,141,142^. In the study by Al-Shakry and his colleagues^140^, two polymer solutions that had undergone pre-shearing experienced a considerable degradation in the range of 16%–30% from their initial bulk viscosity. This resulted in a significant reduction, exceeding 50%, in their resistance factor and viscoelastic properties when they were introduced into Bentheimer rock. A similar pattern emerged when the polymers were injected into Berea cores. In this case, the pre-sheared polymer solution exhibited lower resistance factors, and the onset of shear-thickening occurred at higher velocities compared to the pre-filtered solution. Moreover, several studies have demonstrated that polymer pre-shearing improves polymer injectivity, particularly in low-permeability environments^52,140,143^. The aforementioned phenomenon may be attributed to the decrease in the molecular size of the polymer as a result of pre-shearing, leading to a reduction in its elasticity and an improved propagation through porous media. However, the injectivity improvements must be weighed against the reduction in apparent viscosity^141^.

In summary, polymer molecular weight and injection rate can improve polymer viscoelastic properties. Also, polymer concentration and degree of hydrolysis have a similar positive effect; however, it vanishes beyond a threshold value. The effects of solution salinity and hardness remained inconclusive. It is widely accepted that the viscoelasticity of conventional HPAM polymers deteriorates with increasing concentrations of monovalent and divalent cations in the brine. However, it was also shown that the elasticity of dilute HPAM solutions could be considerably improved with solution salinity. Additionally, several polymers with improved structural chemistry may also show excellent shear-thickening behavior at elevated salinities. It has been also discovered that the addition of nanoparticles to the polymer solution can enhance the viscoelastic properties of polymers. Furthermore, it was found that permeability and temperature have a negative impact, so the polymer viscoelasticity is weaker in high-permeable rocks or under extreme temperatures. Residual oil saturation has a dual effect on polymer viscoelastic behavior. It was found that the presence of oil during in-situ measurements significantly reduces the degree of polymer shear-thickening, but it accelerates the dilatant regime and shifts the onset value to lower rates. Finally, shearing of polymers before injection shifts the onset of dilatancy to higher rates and reduces the degree of polymer shear-thickening. These factors are listed in Table 3.

**Table 3:** Factors controlling polymer viscoelasticity (positive refers to the viscoelasticity increment, while negative is its decrease).

No	Factors	Effect on Polymer Viscoelasticity
1	Polymer Molecular Weight (M_w_)	Positive
2	Polymer Concentration (C_p_)	Positive with threshold
3	Temperature	Negative
4	Brine Salinity (TDS)	Inconclusive
5	Brine Hardness	Inconclusive
6	Permeability	Negative
7	Nanoparticles	Positive
8	Residual Oil Saturation	Dual
9	Degree of Hydrolysis	Positive with threshold
10	Injection Rate	Positive
11	Pre-shearing	Negative

## Residual oil saturation reduction by viscoelastic polymers

**Figure 9:**
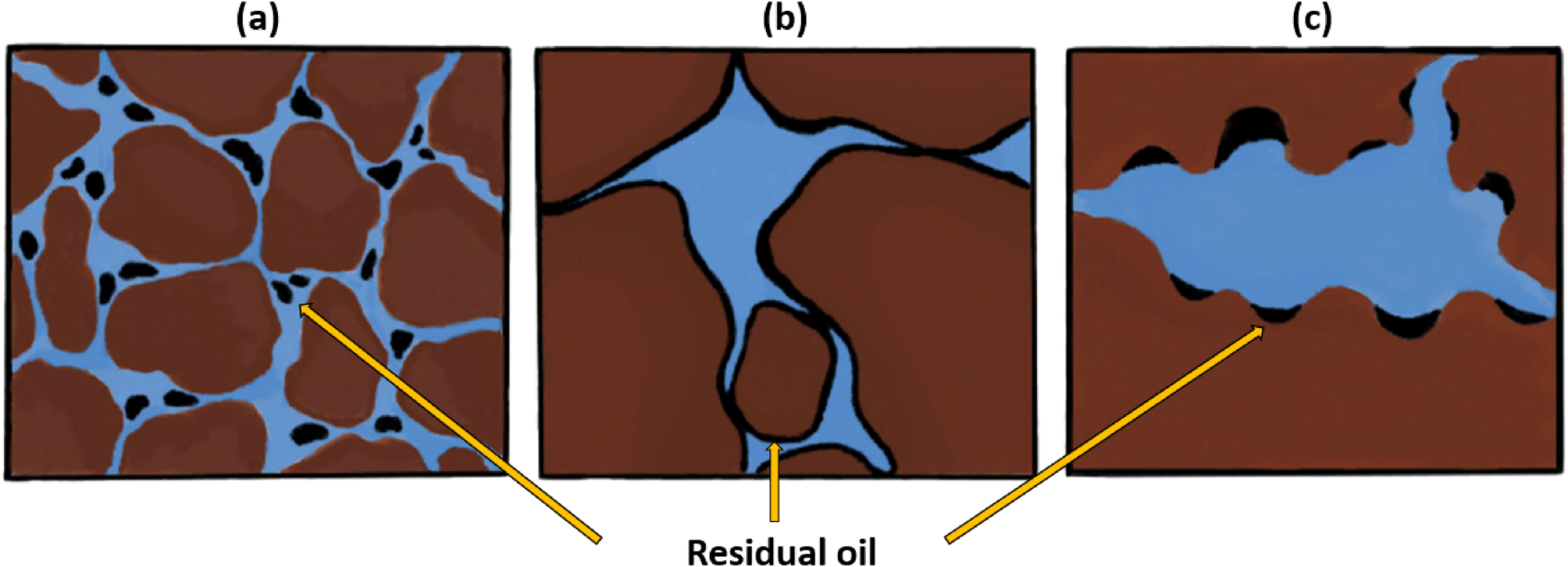
Various types of residual oil in porous media: (**a**) oil ganglia trapped in the pore bodies of a water-wet reservoir due to high capillary forces; (**b**) oil adsorbed on oil-wet rock surfaces; (**c**) immovable oil in stagnant flow pockets. Note that these trapping mechanisms can coexist in porous media.

Residual oil typically refers to the oil that remains trapped in porous media after conventional recovery techniques. There are various factors inducing oil entrapment. For instance, capillary pressure is the primary reason for oil immobilization, particularly in water-wet reservoirs. In such reservoirs, during a waterflood operation before reaching the S_orw_ point, the oil phase flows through a network of interconnected pores in the form of channels or columns. These columns are surrounded by a thin water layer along the pore walls. As the water saturation level increases, the continuous oil column gradually shrinks and breaks into smaller oil droplets, or ganglia, which then get trapped in porous media due to high capillary forces. This phenomenon, commonly known as the "snap-off" of the oil column into oil ganglia, takes place when there are minor deformations in the water/oil interface, resulting in a reduction of interfacial energy. It is important to mention that some researchers call this capillary-trapped residual oil as bypassed oil^144,145^. In contrast, we use the term "bypassed oil" to refer to the mobile oil that remained bypassed in porous media after waterflooding due to reservoir heterogeneities or mobility contrast (Fig. 1). Furthermore, another mechanism substantially increasing residual oil saturation, particularly in oil-wet reservoirs, is the adsorption of oil molecules on the rock surfaces. Finally, the rock configuration might be another factor leading to a large amount of oil trapped in stagnant parts of a reservoir^20,146–148^. These oil-trapping mechanisms are illustrated in Fig. 9.

Generally, residual oil mobilization can be evaluated by comparing capillary and drive forces acting on oil globules. As mentioned earlier, capillary forces may result in disconnected and immobilized oil ganglia in porous media during immiscible fluid displacement^77^. On the contrary, viscous forces applied through injection fluids and possibly gravity forces may mobilize the entrapped oil if they locally balance and exceed the capillary forces. In this case, the contribution of gravity forces to oil mobilization strongly depends on the direction of displacement and the density difference between displacing and displaced fluids^14^. It is important to note that the viscous forces are controlled by the applied pressure drop, the permeability of the porous medium, and the viscosity of displacing fluid. On the other hand, capillary forces depend on the pore geometry, the interfacial tension between two fluids, and the wettability of the rock^149^.

**Figure 10:**
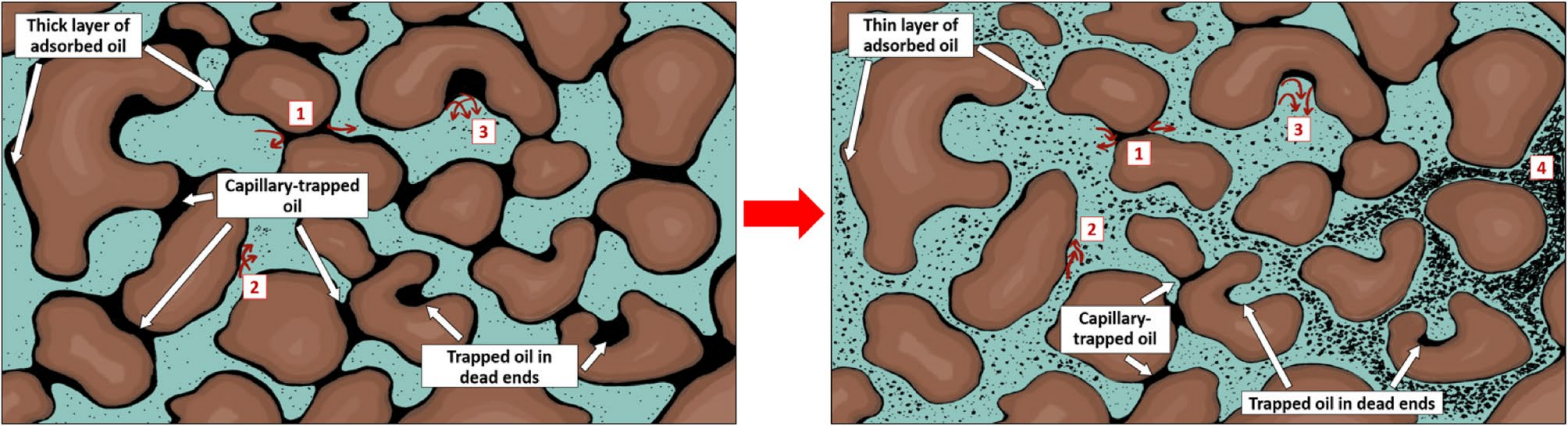
Various mechanisms mobilizing the trapped oil by viscoelastic polymers: (1) the viscoelastic polymers "pull" the oil droplet entrapped in the narrow pores by capillary forces, (2) the oil film adsorbed on the rock surfaces is "stripped off" by viscoelastic polymers, (3) the oil accumulated in stagnant pockets is also "pulled" by viscoelastic polymers, (4) the mobilized oil droplets are dragged to the flow channels in the form of oil threads gradually forming an oil bank.

Conventionally, polymer flooding was associated only with the production of bypassed oil and improvement in macroscopic sweep efficiency, while its effect on residual oil displacement at a micro-scale was thought highly questionable^77,148^. Recent research and field data indicate that viscoelastic polymers may indeed enhance both macroscopic and microscopic efficiencies, contrary to traditional belief. In viscoelastic polymers, the elastic forces represent an essential "pulling" mechanism in mobilizing residual oil, besides the viscous and gravity-driven forces mentioned above. The reduction of residual oil saturation by viscoelastic polymers is hardly obtained by exceeding the critical capillary forces, and the trapped oil mobilization is primarily achieved by stripping off, pulling, and dragging the immobile oil molecules to the flow channels^16,20,90,150^ (Fig. 10). In this context, the stripping-off mechanism aids in reducing the thickness of the adsorbed oil film on the pore walls. When a fluid with the optimal viscoelastic properties moves within capillary walls, it generates a velocity gradient. Consequently, a strong force is produced as the fluid flows through the rock pores, resulting in the removal of the adhered oleic phase from the surfaces of the porous medium^11,135,151^. Furthermore, the elastic effects become more pronounced in the high-shear rate sections of a reservoir, particularly in the vicinity of the injection wells, where severe elongational deformation and elastic turbulence result in substantial extensional viscosities and normal forces^17,152^. The normal forces are closely associated with polymer elasticity and dramatically increase with Deborah Number. It is noteworthy that these elastic micro-forces dominate over viscous forces during highly viscoelastic flow with high $De$ and are the primary reason for oil mobilization^146,147^.

### Literature review on polymer viscoelastic effects on displacement efficiency

As mentioned earlier, the positive effect of viscoelastic polymers on residual oil mobilization has been observed and confirmed at both lab and field scales^16,18,20,132,153–156^. In particular, the first indication of displacement efficiency improvements at the field scale was indicated by Wang et al.^155^. The authors reported 7%-20% incremental oil recovery achieved by the tertiary polymer flooding following extensive waterflooding in the Daqing oilfield. Additionally, several experimental studies investigated the potential of viscoelastic polymers to reduce residual oil saturation. Wang et al.^20^ thoroughly studied various types of residual oil in porous media and revealed how viscoelastic polymers could mobilize that entrapped oil, improving displacement efficiency. It was recommended to use a low-salinity polymer solution with high polymer concentration and large molecular weight to enhance the elasticity of a polymer and strengthen its impact on oil mobilization. Urbissinova et al.^19^ also studied the effect of viscoelastic polymers on residual oil recovery. To distinguish the impact of elasticity on oil mobilization, they used two polymers with comparable molecular weights but distinct molecular weight distributions (MWD). These polymers had the same shear viscosities, but the one with wider MWD had more prominent elastic characteristics. According to their experimental reports, residual oil recovery was higher for the highly-elastic second polymer with a broader MWD. Moreover, coreflooding experiments performed by Vermolen et al.^157^ in Bentheimer sandstones revealed that highly viscoelastic HPAM polymers could improve displacement efficiency by mobilizing low viscous crude (9 cP). The authors also evaluated other polymers with smaller Deborah numbers but with the same viscosities, and no additional oil was produced. As a result, it was concluded that residual oil reduction was caused by polymer elasticity and not by viscous stripping. However, in the case of highly viscous oil (300 cP), the positive effect of viscoelasticity on oil mobilization vanished since no apparent oil recovery was obtained even at high flow rates and Deborah numbers.

**Figure 11:**
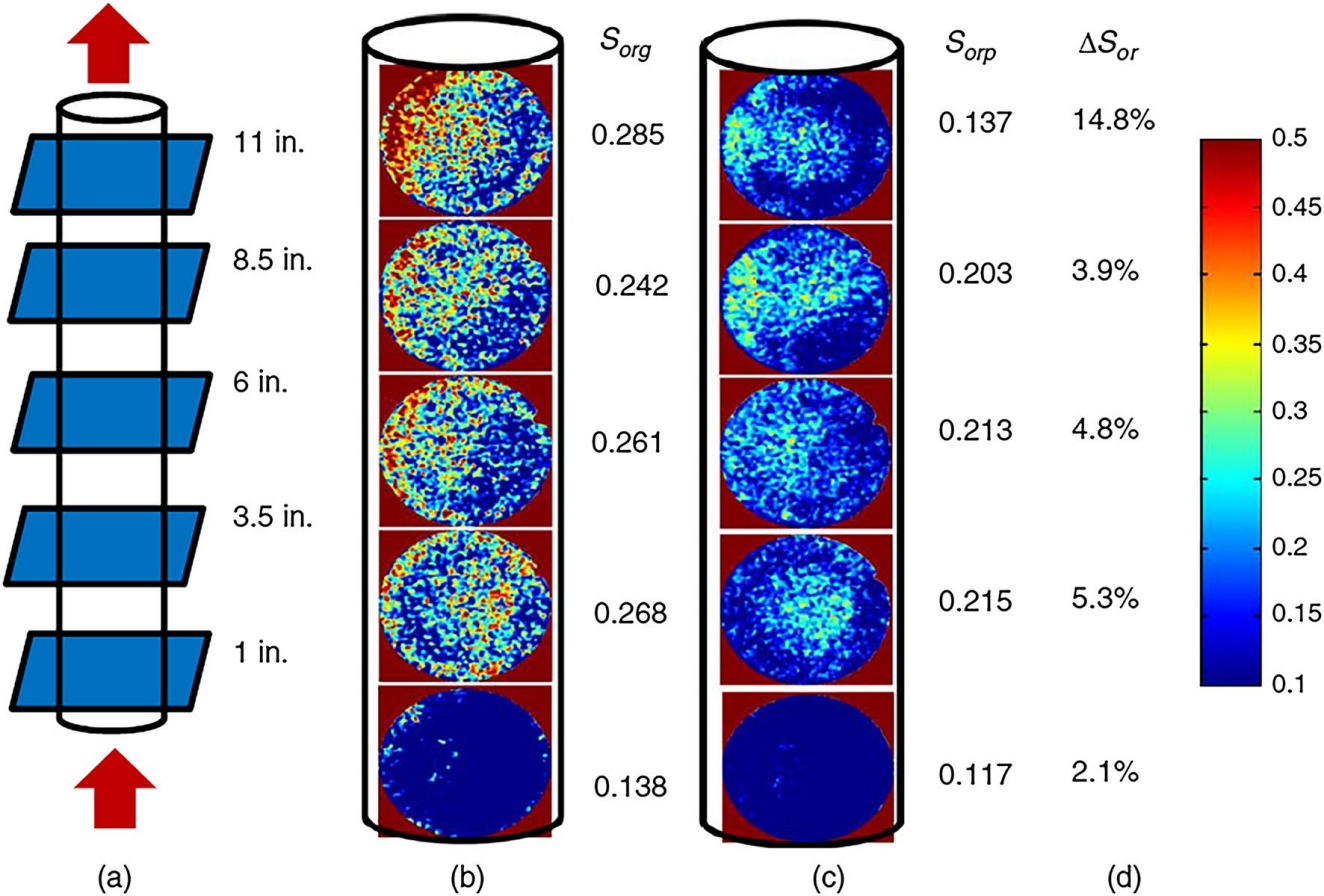
The visualization of residual oil saturation: (**a**) location for 5 CT images along the core, (**b**) the residual oil saturation after glycerin flooding (S_org_), (**c**) the residual oil saturation after viscoelastic polymer flooding (S_orp_), (**d**) the calculated residual oil saturation reduction at each location, which is comparable to a total of 5% OOIP of oil recovery from effluent samples^18^.

Another study investigating residual oil saturation reduction by viscoelastic polymers was performed by Qi et al.^18^. In contrast to the previous research by Vermolen et al.^157^, their coreflooding experiments in Bentheimer sandstones saturated with heavy oil (120 cP) revealed a substantial incremental oil recovery by the viscoelastic HPAM polymers over purely viscous fluids. In particular, viscoelastic polymer flooding was performed at a tertiary mode following water and glycerin (purely viscous) injection to ensure the true residual oil was achieved before starting polymer flooding. Figure 11 illustrates the residual oil distribution in one of their coreflooding experiments conducted with a CT scanner. According to the figure, the residual oil saturation along the core was considerably reduced by injecting elastic polymers after glycerin flow. It should be noted that the trapping number was set substantially below the critical value for desaturation during all coreflooding experiments. Producing oil at the trapping numbers from the flat portion of the capillary desaturation curve (CDC) confirmed that viscoelastic polymers can mobilize residual oil even without exceeding the critical capillary pressure. Trapping numbers and CDC will be discussed in more detail later.

Continuing the exploration of viscoelastic polymer flooding effects on residual oil saturation, it is important to consider some surprising results from Erincik et al.^153^, highlighting unexpected outcomes in tertiary polymer flooding experiments involving variations in salinity conditions. They performed tertiary polymer flooding tests in Bentheimer sandstones by successively injecting low-salinity and high-salinity polymer solutions. The initial slug prepared at low-salinity conditions had a higher oscillatory Deborah number and decreased the residual oil saturation over secondary viscous flow. However, considerable residual oil saturation reduction was also detected even after the subsequent poorly viscoelastic polymer slug, which was prepared at high salinities and had much lower oscillatory Deborah numbers. Such unexpected results were difficult to explain, considering that both polymer solutions had the same viscosities and were injected at the trapping numbers lower than the critical value for desaturation. According to Azad and Trivedi^128^, it is not reasonable to evaluate polymer viscoelasticity based on oscillatory measurements, which cannot accurately reflect the impact of salinity on oil recovery. They measured the extensional relaxation times of different polymer solutions and reported that salinity could actually improve polymer viscoelasticity and oil recovery. However, there might be other explanations for the unexpected results by Erincik et al. In particular, considerable oil production by high-salinity polymers after highly viscoelastic low-salinity polymer flooding might be because of chemical interactions with the clay particles in the porous medium. It might lead to fine migration and clay swelling during high-salinity polymer injection and eventually improve oil recovery at the end of the second polymer slug^64^. An alternative explanation might be the secondary trapping of mobilized oil during the first viscoelastic polymer flood. This oil can be partially or entirely recovered by the second polymer cycle. However, further investigations are needed to prove whether salinity can actually improve viscoelastic behavior or if other factors are involved in such experiments.

It is not always straightforward to distinguish whether oil recovery by tertiary viscoelastic polymers is genuinely due to microscopic displacement efficiency improvements or if it is the production of the oil bypassed during secondary recovery. In other words, sweep efficiency increment might be the underlying reason behind oil recovery at the tertiary stage if specific experimental procedures are not followed to achieve the true residual oil saturation to water. For instance, Koh et al.^96^ conducted several coreflooding experiments utilizing homogenous Ottawa and reservoir sands saturated with viscous oils. They observed a significant oil recovery by the tertiary polymer flooding following extensive water injection. However, the fractional-flow analysis revealed that this recover was primarily due to the production of bypassed oil and macroscopic sweep efficiency improvements. Despite the homogeneity of the cores and extensive secondary waterflooding, it became apparent that the true residual oil saturation was not achieved before polymer injection. The authors reported that the actual reduction in residual oil saturation by viscoelastic polymers was less than 0.03 for most of the coreflooding experiments. Additionally, the tests conducted by Ehrenfried^147^ were also found inconclusive in proving that viscoelastic polymers may diminish residual oil saturation in water-wet sandstones. Although some experiments have confirmed the existence of an elastic effect, other tests either invalidated the premise or demonstrated little to no difference between elastic and non-elastic displacement fluids. According to Guo et al.^158^, the elastic effects hypothesis also could not be ruled out in most circumstances, and there might be other reasons for the increased oil recovery. In this regard, they revealed that the additional oil recovery by viscoelastic polymers might also be related to the changes in rock wettability or to particular experimental artifacts. The capillary end effect is one of these experimental artifacts leading to the misinterpretation of residual oil, particularly in heterogeneous carbonate rock samples^159^. It is frequently observed that performing coreflooding experiments at reservoir rates (1 ft/day) with highly permeable core samples (> 100 mD) leads to a considerable amount of mobile oil left in the rock because of capillary end effects^160^. In order to avoid the mentioned artifact and reach true residual oil saturation, it is essential to conduct the waterflooding tests at high bump rates or to use centrifuge experiments for imbibition instead of coreflooding tests^161,162^. Estimating a true residual oil saturation after waterflooding (S_orw_) is critical in evaluating the effect of tertiary viscoelastic polymer flooding on displacement efficiency.

Continuing from the complexities of residual oil saturation assessment, it is imperative to investigate the merits of implementing polymer flooding during the secondary or tertiary modes, a topic of utmost importance in the context of optimizing enhanced oil recovery methodologies. Numerous studies have consistently shown that employing polymer flooding during the secondary phase of operations can lead to significantly higher oil recovery compared to its application in post-waterflood scenarios^163–167^. Notably, Needham and Hoe^165^ conducted research in this regard and revealed that secondary polymer flooding has the potential to yield roughly four times the recovery achieved through tertiary polymer flooding operations. According to their findings, the optimal timing for polymer flooding is during the early stages of a waterflood when the water-to-oil ratio (WOR) is less than 10. These results were attributed to the presence of relatively high mobile oil saturations and relative permeabilities at lower WOR values. Similar conclusions were drawn by Huh and Pope^163^, who observed that while polymer flooding is seemingly ineffective in mobilizing waterflooding residual oil at the tertiary stage, its introduction into a rock containing mobile oil can effectively diminish the residual oil saturation to a level lower than that attained through waterflooding alone. This outcome is attributed to the elasticity of the polymer, which limits the "snap-off" mechanism. Consequently, the application of elastic polymers during the secondary stage can minimize disruption and deformation of the oleic/aqueous interface, resulting in thinner or longer oil ganglia. Oil ganglia with those shapes are more likely to be mobilized due to the increased pressure gradient resulting from viscous forces across a ganglion, which can overcome the capillary forces trapping the ganglion at the pore throat^168^. Additionally, a simplified stability analysis conducted by Huh and Pope suggested that the thinning of the oil column may be a more crucial mechanism for reducing residual oil saturation than breaking the oil column into longer lengths. In essence, the fraction of oil trapped within porous media during waterflooding can be effectively recovered if the waterflooding technique is replaced by polymer flooding at the secondary stage. This is because secondary polymer flooding has the ability to hinder the "snap-off" of oil molecules. Conversely, the reduction of residual oil after waterflooding becomes notably challenging when polymer flooding is introduced during the tertiary mode, a point at which those oil molecules are already trapped within the porous media. This discrepancy may help clarify the variations in oil recovery observed during viscoelastic polymer flooding at different stages of the process.

Nevertheless, even though it is widely acknowledged that secondary polymer flooding typically leads to higher oil recovery compared to tertiary polymer injection, there are researchers who have put forth a contrasting view. For instance, Doorwar and Mohanty^169^ conducted coreflooding experiments using sandstone and vuggy dolomite cores saturated with 200 cP crude oil. They also carried out additional pore-scale investigations employing 2D glass micromodels. Surprisingly, their findings indicated that tertiary polymer floods, conducted after waterfloods with viscous oil, resulted in a greater oil recovery when compared to secondary polymer floods in both vuggy dolomite cores and Berea cores. In one of their experiments involving a vuggy dolomite core, the oil recovery reached approximately 32% of the original oil in place (OOIP) at the end of the waterflood stage. Subsequently, the tertiary polymer flood contributed an additional 21% OOIP, resulting in a cumulative oil recovery of 53%. In contrast, the cumulative oil recovery during the secondary polymer flood amounted to only 36% OOIP. In other words, the tertiary polymer flood outperformed the secondary polymer flood by recovering approximately 17% more oil. This enhanced performance of the tertiary polymer flood can be attributed to the vuggy nature of the dolomite. Similar unexpected results were also observed in Berea cores, despite contrary findings reported in existing literature. These outcomes could be associated with the presence of substantial mobile oil that remained bypassed after waterflooding, owing to viscosity contrast. In such cases, tertiary polymer flooding may surpass the effectiveness of secondary polymer injection.

Furthermore, the positive effect of viscoelastic polymers on displacement efficiency in sandstones has been observed in most studies. However, several uncertain aspects still need to be clarified to define the limiting factors for viscoelasticity and to understand how to enhance the corresponding impact on displacement efficiency. These might include investigating other potential mechanisms affecting oil recovery during viscoelastic flooding, further analyzing and comparing oscillatory and extensional measurements, and introducing the most efficient experimental procedures to obtain the optimum viscoelasticity under particular experimental conditions. Another essential aspect that needs to be discussed is that utilizing traditional polymers in carbonates does not result in significant residual oil production. It is associated with extreme reservoir conditions in carbonate rocks, which can degrade the polymer and deteriorate its viscoelastic parameters. Therefore, as expected, poorly viscoelastic polymers with low Deborah numbers cannot reduce the residual oil saturation. However, as mentioned earlier, novel polymers have been recently investigated in carbonates. Although they exhibit promising potential to withstand harsh reservoir conditions, their viscoelasticity and respective impact on displacement efficiency are not clearly understood, and further studies are required in this area.

There are some recommendations regarding viscoelastic polymer flooding in carbonates that can be found in the literature. In particular, several authors highlighted the advantages of combining viscoelastic polymer injection with low-salinity waterflooding (LSW) or engineered waterflooding (EWF)^170–172^. Current emphasis has been focused on EWF and LSW as effective EOR techniques. Fine migration, mineral dissolution, multicomponent ion exchange, and wettability modification are the key processes during EWF or LSW in carbonates that contribute to incremental oil recovery^142,173–177^. Hybrid-engineered water-polymer flooding (EWPF) might mitigate polymer chemical degradation and retention in porous media, enhancing polymer viscoelastic behavior and boosting oil recovery^171,172^.

Another advanced method for enhancing microscopic displacement efficiency using viscoelastic polymers in challenging reservoir conditions involves incorporating nanoparticles into the polymer solution. The introduction of nanoparticles improves the stability of the viscoelastic polymer solution, reducing the risk of premature degradation^59–63^. Furthermore, it has been observed that nanoparticles play a significant role in mobilizing and displacing residual oil, primarily through the alteration of wettability^57,135^. In a study conducted by Maghzi et al.^60^, they demonstrated that the dispersion of silica nanoparticles in a polyacrylamide solution led to a change in the micromodel's wettability, shifting it from being oil-wet to water-wet due to the hydrophilic nature of the selected silica nanoparticles. Additionally, these nanoparticles substantially increased the viscosity of the nanosuspension due to ion–dipole interactions between cations and silica, further contributing to enhanced oil recovery^59^. The mechanism of wettability alteration by nanoparticles was also validated by Rahimi et al.^136^. They captured and analyzed images of micromodel pores after each experiment to assess the polymer performance in driving residual oil through the reservoir pores. Their findings indicated a notable reduction in the thickness of the displaced oil layer on the pore wall after the clay polymer nanocomposites (CPN) flooding compared to conventional waterflooding. When the polymer and nanoclay came into contact with the residual oil, the oil droplets transformed into emulsions within the solution. These tiny oil droplets were dragged into flow channels. Furthermore, these droplets coalesced to form an oil front as they converged along their pathways ahead of the injected solution bulk^136^. In summary, the addition of nanoparticles to viscoelastic polymer solutions improves stability, modifies wettability, and enhances oil recovery by mobilizing and displacing remaining oil. Consequently, it is not surprising that this method is gaining recognition as an innovative and increasingly favored technique for significantly enhancing microscopic displacement efficiency in challenging reservoir conditions, sparking significant interest in the field of enhanced oil recovery.

### Overview of the analytical approaches to quantify residual oil saturation

#### Trapping number

The trapping number (${N}_{T}$) is a crucial factor that controls the gravity and viscous effects on residual oil recovery^178^. It can be computed through the Bond (${N}_{B}$) and capillary numbers (${N}_{C}$), as given in Eq. (9).^179^ In this case, the Bond number links gravity and capillary forces, while the capillary number represents the relationship between viscous and capillary forces.

9 $${N}_{T}=\sqrt{{N}_{B}^{2}+2{N}_{B}{N}_{C}\mathrm{sin}\alpha +{N}_{C}^{2}} ,$$

10 $${N}_{B}=|\frac{{k}_{{p}^{\prime}}g\left({{\rho }_{{p}^{\prime}}-\rho }_{p}\right) }{{\sigma }_{{p}^{\prime}p }cos\theta }| ,$$

11 $${N}_{C}=|\frac{{k}_{{p}^{\prime}}\cdot \nabla {\Phi }_{p^{\prime}} }{{\sigma }_{{p}^{\prime}p }cos\theta }| ,$$

12 $${\Phi }_{p^{\prime}}={P}_{p^{\prime}}-g{\rho }_{p^{\prime}}\Delta D ,$$

where $\alpha$ indicates the flow direction with respect to positive x axis, ${k}_{{p}^{\prime}}$ is the effective permeability of displacing fluid, $\theta$ is the contact angle, ${\sigma }_{{p}^{\prime}p}$ is the interfacial tension between two phases, ${\Phi }_{p^{\prime}}$ is the flow potential of displacing phase, $g$ is the gravitational constant,${P}_{p^{\prime}}$ is the pressure of the displacing phase,$\Delta D$ is the vertical distance between the datum and the position below, and ${\rho }_{{p}^{\prime}}$ and ${\rho }_{p}$ are the densities of displacing and displaced phases, respectively^180^.

The Eq. (9) can be simplified to Eq. (13) for horizontal flow, while Eq. (14) represents the vertical flow:

13 $${N}_{T}=\sqrt{{N}_{B}^{2}+{N}_{C}^{2}} .$$

14 $${N}_{T}=\left|{N}_{C}\pm {N}_{B}\right| .$$

In the latter equation, " + " and "−" are used for vertical upward and downward flow, respectively.

#### Capillary desaturation curve

As shown in the previous section, the capillary number is the ratio of viscous to capillary forces. Initially, Moore and Slobod^181^ introduced the concept of capillary number to explain the mobilization of oil ganglia entrapped in porous media. Subsequently, several modifications have been made to ${N}_{C}$ to improve its correlation with residual oil saturation^159^. Some of those advancements were briefly discussed by Azad and Trivedi^128^. On the other hand, the Bond number provides the quantitative means to estimate the effect of gravity forces on trapped oil recovery. The traditional capillary number method may not accurately predict the oil mobilization if gravity forces are significant. According to Pennell et al.^179^, the vertical displacement in 20–30 mesh Ottawa sand conducted at Darcy velocities below 2.8 ft/day had Bond numbers one order of magnitude higher than capillary numbers. Therefore, using only a capillary number was inadequate to predict the observed oil recovery. Generally, the Bond number’s effect on the trapping number may vary depending on the direction of flow and the density difference between displacing and displaced phases. Eventually, to get a higher trapping number, it is required to maintain the vertical upward flow ($\alpha$ = 90°) for the heavier displacing fluid. On the contrary, it is recommended to have a vertical downward flow ($\alpha$ = -90°) for a lighter displacing fluid^14^.

**Figure 12:**
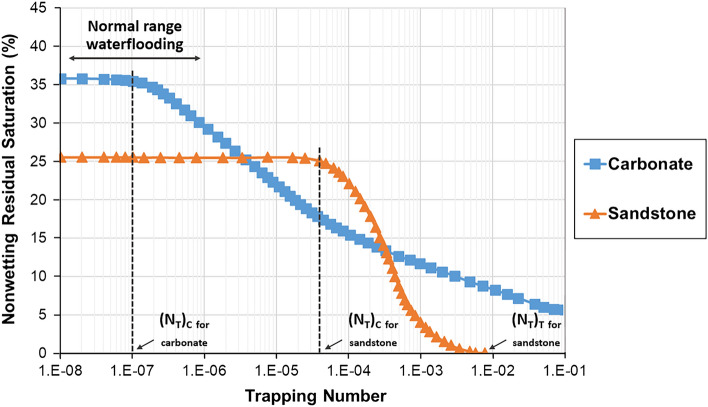
The typical Capillary Desaturation Curve (CDC) for carbonates with a wide pore size distribution and well-sorted sandstones (modified after Lake et al.^71^).

The combined impact of viscous and gravity forces on residual oil saturation reduction can be illustrated in the capillary desaturation curve (CDC), showing the residual saturations of fluids as a function of trapping numbers (Fig. 12). Traditionally, it is believed that the residual phase saturations stay plateaued on a flat portion of CDC until the critical point for desaturation. Furthermore, an increment in trapping numbers beyond this critical value ${\left({N}_{T}\right)}_{C}$ leads to phase mobilization, and the reduction in residual saturations takes place until the complete desaturation, where all trapped phase fluids are mobilized and displaced. The corresponding trapping number is called the total desaturation trapping number ${\left({N}_{T}\right)}_{T}$^14^. It is important to note that several factors can change the shape of the capillary desaturation curve and affect the mobilization of residual phases. These factors include, but are not limited to, rock wettability and heterogeneity. Rock wettability is one of the major factors controlling residual phase saturations. It was reported that the residual oil saturation is the lowest in rocks with neutral wettability, while the highest residual oil saturation can be observed in oil-wet samples due to the strong affinity of the rock surface to oil molecules^159,182^. The reason for this phenomenon lies in the nature of capillary desaturation curves. CDCs of mixed-wet rocks have steeper slopes and different critical capillary number ranges compared to oil-wet and water-wet rocks^183^. Additionally, the oil saturations in mixed-wet and oil-wet samples can be 15–20% higher than in water-wet rocks REF^184^. On the other hand, rock heterogeneity and geometry also play a primary role, dictating the critical trapping numbers and residual phase saturations. For instance, rocks with larger average pore size and smaller ratios of pore body-to-throat size facilitate less challenging mobilization of trapped fluids from the porous medium. Additionally, the pore size distribution is another crucial factor controlling the shape of the CDC^148^. As can be seen from Fig. 12, the carbonates having wide pore size distribution are usually associated with shallower CDC^71^. Conventionally, the shallow CDC of carbonates is related to their low critical trapping numbers (${\left({N}_{T}\right)}_{C}$) and high total desaturation trapping numbers (${\left({N}_{T}\right)}_{T}$)^185,186^. In particular, ${\left({N}_{T}\right)}_{C}$ for carbonates reported in a number of studies ranges from 10^–8^ to 10^–6^, which is at least two orders of magnitude lower than that for sandstone rocks^187,188^. Therefore, it is traditionally believed that inducing oil mobilization and producing some trapped oil is easier in carbonates. However, it is complicated to mobilize and displace the remaining residual oil due to shallower CDC and higher ${\left({N}_{T}\right)}_{T}$ values in carbonate rocks^189^. Nevertheless, in contradiction to the above, several studies reported high critical trapping numbers for carbonates, ranging from 10^–5^ to 10^–4^^160,162,190^. Disagreement among the authors on actual ${\left({N}_{T}\right)}_{C}$ for carbonates can be associated with the various experimental protocols utilized in the corresponding studies. As previously mentioned, it is essential to eliminate the experimental artifacts by conducting coreflooding tests at high bump rates or performing multi-speed centrifuge experiments for desaturation. Selecting the proper experimental procedure may help reach the true residual oil saturation and estimate the actual critical trapping number for carbonate rocks. Therefore, further experimental investigation is required to confirm those high critical trapping numbers in carbonates and to reach a consensus on this topic.

Most sandstones have critical trapping numbers ranging from 10^–5^ to 10^–4^. On the other hand, the typical trapping numbers achieved during waterflooding at a Darcy velocity of 1 ft/day and effective aqueous permeability of 100 mD usually vary in the range of 10^–7^–10^–6^^146^. Since these trapping numbers are below the critical desaturation values for sandstone, it is believed that water injection cannot reduce the non-flowing oil saturation in this type of rock ^6^. Therefore, introducing new methods to increase the trapping numbers is a must in order to mobilize the residual oil saturation. Several techniques to boost trapping numbers are summarized in Table 4^6,8,21,191^. According to the table, one way to decrease residual oil saturation is by reducing IFT between aqueous and oleic phases using a surfactant or alkaline flooding. This method can increase the trapping numbers by several orders of magnitude and efficiently improve microscopic displacement efficiency. On the other hand, polymers may increase injectant viscosity from around 1 cP to 10–100 cP and boost trapping numbers several times. However, it was believed that even this increase provided by purely viscous polymers cannot lead to oil mobilization in sandstones, explained by the polymer flooding trapping numbers being still lower than the critical trapping number for desaturation. Therefore, polymers were commonly thought to affect macroscopic sweep efficiency only.

**Table 4:** Techniques increasing trapping number.

No	Methods	Comments
1	An increment in displacing fluid viscosity	It can be achieved by adding polymers to the injected fluid
2	A decrease in interfacial tension (IFT) between displaced and displacing fluids	Surfactants or alkaline flooding might reduce IFT between oleic and aqueous phases from about 30 to 10^–4^–10^–3^ mN m^−1^
3	An increase in the displacing fluid velocity, and correspondingly in the injection pressure	It is essential to consider a formation fracture pressure while incrementing the injection rate to avoid excessive fracturing
4	A reduction in IFT and increment in viscosity simultaneously	This task can be attained by utilizing viscoelastic surfactants or surfactant-polymer (SP) flood
5	A wettability alteration towards neutral wettability ($\theta \to {90}^{0}$)	Can be achieved by using surfactants or adjusting the injection water chemistry
6	Enhancing the contribution of gravity forces	Increasing the density difference between the phases and choosing an efficient displacement trajectory

However, it is interesting to note that recent studies are consistent with the idea that viscoelastic polymers can reduce residual oil saturation to water through their elastic properties. It was found that oil mobilization by viscoelastic polymers can take place even at low trapping numbers below the critical values^16,18,107,108,153^. Therefore, the traditional capillary desaturation curves may not correctly represent oil recovery by viscoelastic polymers. Substantial extensional viscosities and normal forces attributed to the high Deborah numbers explain the displacement of residual oil by viscoelastic polymers. Accordingly, synthetic polymers with viscoelastic properties increase both microscopic and macroscopic efficiencies^189^.

As mentioned earlier, traditional CDC is insufficient to properly evaluate the increment in microscopic displacement efficiency by viscoelastic polymers since it incorporates the effects of viscous and gravity forces only. In contrast, the main mechanism of viscoelastic polymers to mobilize residual oil in porous media is elastic "pulling" forces. Therefore, several modification and adjustments of CDC were made to accommodate these elastic effects for viscoelastic polymers. The following section will give insights into the calculation of residual oil saturation using trapping and Deborah numbers and introduce the modified capillary desaturation curves provided in the literature.

#### Estimation of residual oil saturation during viscoelastic polymer flooding

As discussed in the previous section, conventional CDC analysis may not be sufficient to reveal actual residual oil saturation during viscoelastic polymer flooding. This is because viscoelastic polymers exhibiting a complex behavior in porous media can displace residual oil even at low trapping numbers through their elastic recovery mechanisms. This section summarizes some of the recent models estimating residual oil saturation. These models listed in Table 5 are discussed below.

**Table 5:** List of models predicting residual oil saturation.

No	Models	References
1	${S}_{or}={S}_{or1}^{high}+\frac{{S}_{or1}^{low}-{S}_{or1}^{high}}{1+{T}_{1}{N}_{T}^{\tau }}$	Delshad^192^
2	${S}_{or1}={S}_{or1}^{high}+\frac{{S}_{or1}^{low}-{S}_{or1}^{high}}{1+{T}_{1}{N}_{T}}$ ${S}_{or2}={S}_{or2}^{high}+\frac{{S}_{or2}^{low}-{S}_{or2}^{high}}{1+{T}_{2}De}$ ${S}_{or}=\mathrm{min}({S}_{or1},{S}_{or2})$	Lotfollahi et al.^193^
3	$${S}_{or}^{*}=\left\{\begin{array}{l}1 \,if \,De<1 \\ 1-0.133logDe \,if \,De\ge 1\end{array}\right.$$	Qi et al.^139^
4	$${S}_{or}=\left\{\begin{array}{l}-0.009\mathrm{ln}\left({N}_{ce}\right)+0.3348\, if\, {N}_{ce}<{N}_{crt} \\ 0.287\mathrm{exp}\left(-3.42*{N}_{ce}\right) \,if\, {N}_{ce}>{N}_{crt}\end{array}\right.$$ ${N}_{ce}=\frac{V*{\mu }_{pore}}{{\sigma }_{ow}}$	Azad and Trivedi^128^

Where ${S}_{or}$ is the residual oil saturation, ${T}_{1}$ is the trapping fitting parameter, $\tau$ is the model parameter dependent on the rock pore size distribution, ${S}_{or1}^{low}$ and ${S}_{or1}^{high}$ are the residual oil saturations at critical and total desaturation trapping numbers, respectively;${S}_{or1}$ and ${S}_{or2}$ are the residual oil saturations determined by trapping and Deborah numbers, respectively, $De$ is the Deborah number, ${T}_{2}$ is the viscoelastic fitting parameter (usually taken as 0.3–0.5 in sandstones), ${S}_{or2}^{low}$ and ${S}_{or2}^{high}$ are the residual oil saturations at low Deborah numbers (waterflooding $De$) and high Deborah numbers (viscoelastic polymer flow $De$), respectively,${S}_{or}^{*}$ is the normalized residual oil saturation that is the ratio of residual oil saturation after polymer flooding to the one before polymer injection,${N}_{ce}$ is the extensional capillary number, ${N}_{crt}$ is the critical capillary number,$V$ is the interstitial velocity, ${\sigma }_{ow}$ is the interfacial tension between oleic and aqueous phases, and ${\mu }_{pore}$ is the pore-apparent viscosity that is calculated using extensional parameters.

The well-recognized model proposed by Delshad^192^ was originally used to construct the conventional CDC for aqueous, oleic, and microemulsion phases. Although this model cannot adequately evaluate residual oil saturation by viscoelastic polymers, it can still be utilized for biopolymers with poor viscoelastic responses in porous media. Moreover, Lotfollahi et al.^193^ introduced a slightly modified model, where the authors tried to comprehensively accommodate the impact of polymer elasticity on oil mobilization. They proposed calculating residual oil saturation using both trapping and Deborah numbers and considering the lowest of these two as the actual residual oil saturation. Furthermore, the oil recovery and pressure drop data from secondary and tertiary polymer flooding experiments were effectively history-matched using the given model. However, it might be improved further to account for a broader range of fluid and rock properties.

**Figure 13:**
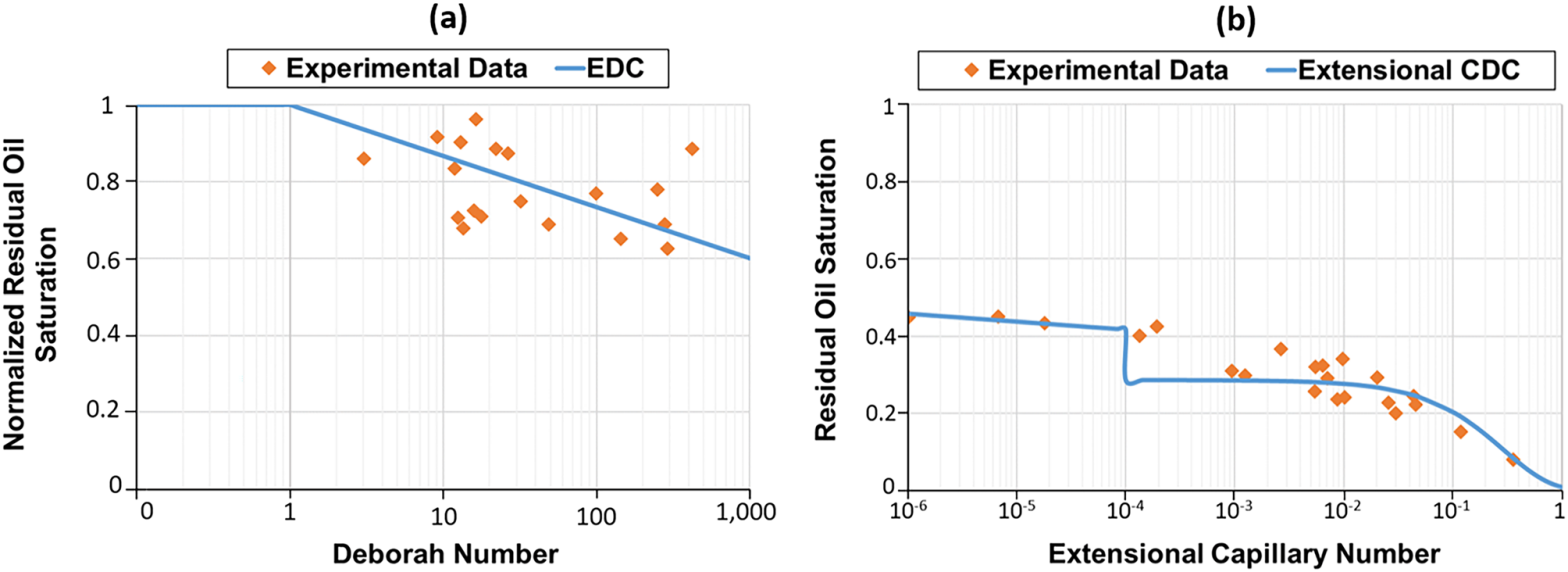
Estimation of residual oil saturation using various models: (**a**) Elastic Desaturation Curve (EDC) (modified after Qi et al.^139^), (**b**) Extensional Capillary Desaturation Curve with the critical capillary number of 10^–4^ (modified after Azad and Trivedi^128^).

Furthermore, Qi et al.^139^ provided an alternative to the capillary desaturation curve by correlating the residual oil saturation reduction to the Deborah numbers and named it as Elastic Desaturation Curve (EDC) (Fig. 13a). According to their model, no oil mobilization can be observed at low Deborah numbers. In contrast, the residual oil saturation may be substantially reduced during highly elastic polymer flooding with large Deborah numbers. Moreover, polymer relaxation times and shear rates were the primary factors controlling polymer elasticity and oil recovery predictions. EDC was successfully used to history-match the experimental pressure drop and oil recovery data. Additionally, core-scale and field-scale simulation studies revealed that reservoir heterogeneity and permeability, polymer concentration, brine salinity, oil viscosity, well pattern size, and the injection rate could considerably affect the displacement of residual oil by viscoelastic polymers. However, Azad and Trivedi^128^ pointed out that the prior model by Qi et al. failed to estimate the residual oil saturation for different salinity conditions. As a solution, they introduced a novel model based on the extensional capillary number (Fig. 13b). It was found that the residual saturations predicted by the extensional model were closer to actual values for the high-salinity solutions. However, the parameters (${n}_{2}$, ${\tau }_{ext}$, ${\mu }_{max}$) determined by an extensional rheometer lead to very high pore-apparent viscosities. In several experiments utilizing viscoelastic polymers, the observed polymer viscosities ranged from 1,000 to 500,000 cP, which is unreasonable for practical field applications.

Finally, it is important to note that the reported models estimating residual oil saturation are mainly derived and correlated for sandstones. Therefore, it is crucial to perform similar studies in carbonates and quantitatively evaluate the displacement efficiency in this type of rock. It is crucial to consider the harsh conditions in most carbonate reservoirs, including high salinities. Therefore, it is recommended to establish an unambiguous relationship between polymer viscoelasticity and salinities first to obtain more accurate oil recovery predictions in these rocks. Subsequently, the residual oil saturation in carbonates after viscoelastic polymer flooding may be computed either by adjusting the fitting parameters in the existing models or generating new and more comprehensive models.

## Challenges faced during viscoelastic polymer flooding

### Mechanical degradation

One of the primary issues faced during viscoelastic polymer flooding is mechanical degradation which may severely impair polymer viscosifying and elastic power^194^. This process often takes place in polymer handling equipment, chokes, pumps, perforations, and in a wellbore vicinity^111,195^. Moreover, the synthetic viscoelastic polymers are more sensitive to degradation than biopolymers^27^. The flexible random-coil structure of the synthetic polymers is reported as the underlying reason behind this rapid degradation^74^. Such conformation usually results in the shear-thickening behavior of viscoelastic polymers, where significant elastic forces are exerted on the polymer backbone, stretching it apart. At shear rates beyond the critical rate for mechanical degradation, these elastic forces overcome macromolecular bonding forces, breaking the polymer backbone and causing a significant viscosity loss^104,196^.

Furthermore, various factors controlling the degree of mechanical degradation in porous media are listed in Table 6^197,198^. Formation permeability is one of the critical parameters; tight reservoirs with low permeabilities exacerbate polymer mechanical degradation due to significant stresses encountered in narrow pore throats. Similarly, injecting polymers at high rates also aggravates the degradation process. This issue can be considerably controlled by generating fractures around the wellbore, which increase the permeability in that region and reduce the velocity of the polymers penetrating the rock^199^. In this regard, Martin^200^ found a substantial variation in the extent of mechanical degradation observed in field trials and laboratory experiments. It was explained that coreflooding experiments consider polymer injection directly into the rock matrix. In contrast, the polymer flooding field applications may be performed in the stimulated wellbores with significantly higher permeabilities in the near-wellbore region than in the rock matrix.

**Table 6:** Factors controlling mechanical degradation (positive refers to the degradation increment, while negative is its decrease).

No	Factors	Effect on Mechanical Degradation
1	Permeability	Negative
2	Injection Rate	Positive
3	Polymer Molecular Weight	Positive
4	Polymer Concentration	Inconclusive
5	Brine Salinity	Positive
6	Brine Hardness	Positive
7	Temperature	Positive

Furthermore, polymer molecular weight is another factor accelerating mechanical degradation^56^. Polymers with higher M_w_ are more susceptible to degradation due to increased flow resistance and considerable mechanical stresses^109^. However, Martin^200^ mentioned that despite the degradation being more severe for high molecular-weight polymers, they might achieve higher viscosities in porous media than those with lower M_w_. Additionally, the same detrimental effect on mechanical degradation holds for brine salinity and hardness. Several studies revealed that the degree of shear degradation could be considerably magnified with the monovalent and divalent ion concentrations in both sandstone and carbonate cores^198,200–204^. Moreover, the viscosity loss due to degradation was reported to be higher in the presence of divalent ions^203^. As a solution, softening or dilution of injected water can reduce mechanical degradation extent. On the other hand, the effect of polymer concentration on mechanical degradation was found inconclusive. While Maerker^198^ reported no apparent effect of concentration on the degradation process, Morris and Jackson^205^ and later Martin^200^ claimed that breakdown could be slowed down at higher polymer concentrations. In contrast, Clemens et al.^206^ shared that polymers at higher concentrations are more susceptible to degradation. Therefore, this impact should be investigated further. Finally, it was found that the temperature has an adverse effect on the degradation process, which was explained by polymer bonds being easily ruptured at higher temperatures^207,208^.

Mechanical degradation may impose significant cost limitations on polymer flooding applications and even lead to project failure, particularly in low-permeable and heterogeneous carbonates^209^. Before initiating polymer flow into a porous medium, degradation may be anticipated and reduced if an injection well is designed and equipped appropriately with special flowmeters, pumps, and tubing^29^. Additionally, increasing polymer concentration may compensate for the viscosity loss in insignificant mechanical degradation. However, entirely different approaches must be used to address severe mechanical deterioration^200^. For instance, using horizontal injection wells for polymer flooding, increasing the perforation density in cased-hole completions, generating fractures around the wellbore by injecting above the formation parting pressure, and pre-shearing the polymer solution prior to injection may significantly alleviate mechanical degradation^198,209^. The polymer pre-shearing considers subjecting a polymer solution to high-speed stirring or high-pressure capillary flow to sever longer-chain polymer components while maintaining a comparable mean molecular weight distribution (MWD). Moreover, the pre-sheared polymers with a high molecular weight may retain their original viscosities better than the commercial polymers with a lower molecular weight^210^. Furthermore, pre-shearing the polymers might result in optimum mobility reduction and enhanced injectivity, considering their tolerance to mechanical breakdown.

### Viscoelastic polymer retention

Retention of polymers in porous media is another major issue faced during viscoelastic polymer flooding. It occurs as a result of the interactions between the rock surface and polymer molecules^77,211^. Retention slows polymer front propagation and decreases polymer viscosity in the aqueous phase. Moreover, it can result in considerable polymer loss to the rock surfaces causing permeability damage and reducing the efficiency of polymer flooding projects^27^. If the degree of polymer retention is less than 100 µg/g-rock, it is regarded as low and efficient^212^. Therefore, evaluating the retention levels for a specific field is essential by performing representative experiments at targeted reservoir conditions.

**Figure 14:**
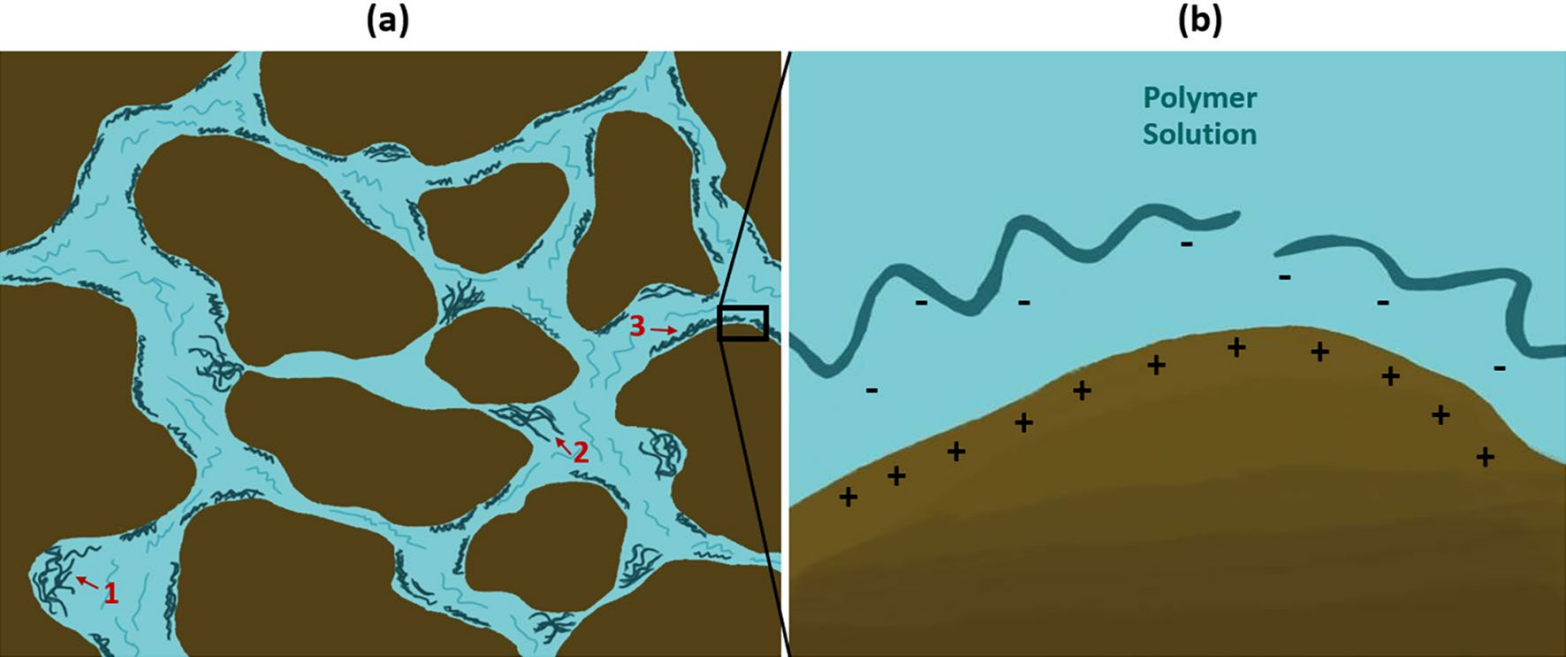
(**a**) various retention mechanisms of viscoelastic polymers in porous media where retained polymers are illustrated with dark blue curves: (1) hydrodynamic polymer retention in stagnant points, (2) mechanical entrapment of polymers in narrow pore throats, (3) polymer adsorption on rock surfaces; (**b**) zoomed-in portion of porous media describes polymer adsorption in a given carbonate reservoir: significant electrostatic interaction existing between Ca^2+^ carrying sites in carbonates and the COO^−^ carboxyl groups in HPAM polymers results in polymer adsorption.

There are three types of polymer retention in porous media based on the governed mechanisms: adsorption, mechanical entrapment, and hydrodynamic retention (Fig. 14)^77,213^. Polymer adsorption is the most prevalent retention type, where polymer molecules attach to the rock surface due to strong attraction forces, occupying the accessible rock surface sites^93^. Therefore, the degree of adsorption rises in the rock with a larger available surface area^214,215^. Moreover, releasing and recovering most of the adsorbed polymers to the surface is very challenging. These polymers, which remain in porous media irreversibly altering and deteriorating formation properties, may reduce the efficiency of polymer flooding projects and other techniques that can be performed subsequently to produce remaining oil after the polymer injection^216^. On the other hand, mechanical entrapment is a different type of polymer retention that occurs when large polymer molecules cannot pass through narrow pore throats^217^. This form of retention is more common in core samples with low to medium permeabilities, and it can be avoided with appropriate polymer filtering. Finally, hydrodynamic retention occurs at high injection rates but has little practical relevance^218,219^.

Various factors may influence polymer retention in a porous media including, but not limited to, permeability, polymer type, molecular weight, rock mineralogy, wettability, and water chemistry^33,215,220^. The parameters affecting polymer retention in porous media are summarized in Table 7. Permeability is a critical factor that influences both mechanical degradation and polymer retention. It was found that the retention is insensitive to formation permeability in high-permeable samples (500 mD), while it can significantly increase with the permeability reduction in tight cores (below 100 mD)^93,221–223^. It is worth mentioning that at low permeability values, mechanical entrapment and hydrodynamic retention become crucial in increasing the degree of polymer retention^199^. Similarly, several studies have demonstrated that the retention of HPAM polymers also increases with increasing polymer molecular weight^33,214^. They also proposed that higher molecular weight polymers adsorbed on a rock surface and generate a thicker layer than lower molecular weight polymers. In addition, it has been observed that viscoelastic polymer retention in carbonate reservoirs is considerably higher than in sandstones because of significant electrostatic interaction between Ca^2+^ carrying sites in carbonates and the COO^−^ carboxyl groups in HPAM polymers (Fig. 14b)^40,212^.

**Table 7:** Factors controlling polymer retention (positive refers to the retention increment, while negative is its decrease).

No	Factors	Effect on polymer retention
1	Permeability (but retention is insensitive to permeability variation above 200 mD)	Negative
2	Polymer Molecular Weight	Positive
3	Calcite and Shale Content	Positive
4	Wettability (towards oil-wet)	Negative
5	Oil Presence	Negative
6	Brine Salinity	Positive
7	Brine Hardness	Positive
8	Polymer Concentration	Inconclusive

Moreover, the wettability state of a rock is another critical factor that may affect polymer retention. Although most laboratory studies investigating polymer flooding in porous media have been conducted in the absence of crude oil, using oil-wet samples can significantly affect polymer performance^224,225^. In particular, polymer adsorption tends to be lower in the presence of oil than in single-phase experiments^40,226–229^. In this regard, some studies reported the reduction in retention to be more than 50% in oil-wet samples^33^. It was related to the fact that most of the adsorbable rock sites were covered by oil molecules during two-phase experiments, reducing the polymer adsorption onto the rock. Furthermore, the effect of water chemistry on retention was also investigated in several studies^228,230–234^. Cationic polyacrylamide (PAM) retention is usually independent of brine salinity and hardness^230^. In contrast, for HPAM applications, it was revealed that monovalent and, particularly, divalent ions in brine can significantly increase polymer adsorption in sandstone samples^231,233^. It can be related to bridging anionic sandstone surfaces and anionic HPAM polymers by calcium ions.

Additionally, the effect of polymer concentration was found to be controversial. It has been shown by some researchers that polymer adsorption/retention is concentration-dependent and fits the Langmuir isotherm^235–237^. On the other hand, some other researchers have claimed that polymer adsorption is concentration-independent^220^. The Langmuir isotherm is a simple model that describes how the adsorption of polymer molecules onto the surface of a rock changes with polymer concentration. The model suggests that polymer adsorption initially increases with an increase in polymer concentration and then slightly reaches a plateau^215,238^. Additionally, the model suggests that polymer retention in the porous medium is reversible. Sorbie^77^ provided a summary of the early studies on the related topic. Similarly, Zhang and Seright^220^ and Kamal et al.^215^ provided reviews about the effect of viscoelastic polymer concentration on the adsorption isotherm. Studies claiming polymer adsorption to be concentration-dependent were mainly based on experiments conducted using sand powders with the static method. Additionally, Langmuir adsorption models have been used to perform simulations of polymer adsorption during polymer flooding^238–240^. Szabo^237^ conducted static adsorption and retention measurements on silica sands and Berea cores. They claimed that the adsorption isotherm of viscoelastic HPAM is higher with increasing brine salinity. Dang et al.^238^ also modeled polymer adsorption by using the Langmuir isotherm theory and found that polymer adsorption is strongly dependent on the polymer concentration. Furthermore, they showed that the adsorption isotherm is also increased by increasing the salinity of the aqueous phase. On the other hand, the experiments conducted using dynamic techniques show that polymer adsorption is concentration-independent and does not fit the Langmuir isotherm. Zhang and Seright^220^ conducted a comparative study of polymer retention using Flopaam 3230S using both static and dynamic techniques by studying polymer retention at various polymer concentrations (10–6000 ppm). They found that polymer adsorption is independent of the polymer concentration used and does not follow the Langmuir isotherm.

Polymer retention can be evaluated by dynamic or static experimental methods^241,242^. The dynamic retention test is the most extensively used approach, which involves injecting a polymer solution into a rock sample and analyzing the polymer content in the effluents. On the other hand, the static method entails combining crushed rock powder with a polymer solution and then testing the supernatant polymer solution for concentration. The static approach overestimates the retention because of the high surface area of the rock powder exposed to the polymer. Moreover, mechanical entrapment retention cannot be captured in static experiments.

#### Literature review on viscoelastic polymer retention in sandstones and carbonates

Most of the reported works focus on polymer flooding in sandstone, with just a little research is reported on carbonate reservoirs. This is owing to a lack of polymers capable of withstanding the severe carbonate reservoir conditions^214^. The use of traditional HPAM-based polymers is limited to low temperatures and salinity conditions. Multiple studies analyzed polymer retention in sandstones. For instance, Zaitoun and Kohler^223^ performed coreflooding tests on sandstone core samples with varying permeabilities to investigate the dynamic retention of HPAM and reported retention values ranging from 100 to 155 µg/g-rock. Similarly, Broseta et al.^226^ performed a number of dynamic retention experiments on sandstones in the presence and absence of oil using HPAM-based polymers. They reported that polymer adsorption was decreased in oil-containing core samples compared to 100% water-saturated core samples. Additionally, Zhang and Seright^220^ conducted static and dynamic retention studies on sandstone outcrop samples using HPAM-based polymers at various concentrations (10–6,000 ppm). They observed polymer retention values ranging from 16 to 56 µg/g-rock in dynamic experiments, while much higher retention values were recorded in static tests. Moreover, the effect of salinity on polymer retention was investigated by Unsal et al.^234^. They conducted several coreflooding experiments using HPAM-based polymers at low and high salinity brine. It was revealed that polymer retention was significantly less in the low-salinity aqueous phase. Furthermore, Wever et al.^229^ used core samples from several sandstone reservoirs to demonstrate the influence of residual oil saturation on HPAM polymer retention. In the presence of oil, they observed low retention values (below 100 µg/g-rock for low permeability samples and 0–11 µg/g-rock for high permeable cores). As expected, polymer adsorption was dramatically increased in the cores with 100% water saturation.

Recent advancements in synthetic polymer manufacturing have enabled polymer EOR in carbonate reservoirs under harsh conditions. The novel polymers include the addition of specific monomers to the chains of polyacrylamides^33,38,40^. Dupuis et al.^33^ experimentally investigated the propagation behavior of a new class of thermally stable polymers in different carbonate core samples. The experiments were conducted to understand the effect of various parameters on the injectivity and adsorption of thermostable polymers (with NVP and ATBS monomer) in Estalliade cores. Dupuis et al. found that Polymer A (containing NVP) has a low adsorption value (22 µg/g-rock) compared to polymer D1 (220 µg/g-rock). However, polymer D1 outperformed polymer A (which showed a constant pressure increase) in terms of injectivity and cost. Hence polymer A was abandoned. The authors further studied the effect of different parameters (salinity, oil presence, and molecular weight) on the performance of polymers D (with high ATBS content) at a temperature of 120 °C. They found that adsorption was not affected by the presence of mineral oil when compared with the cores at 100% water saturation. On the contrary, they observed more than a 50% reduction in polymer adsorption in cores aged for two weeks with crude oil. They concluded that restoring the wettability state was crucial for evaluating polymer retention in porous media.

Masalmeh et al.^40^ conducted polymer injectivity and retention coreflooding experiments on carbonate core samples at high salinity and elevated temperature using an ATBS-based polymer. A polymer concentration of 1,000 ppm was used for all their experiments in core samples with permeability between 29 and 163 mD. The retention was relatively high in the absence of oil (209–316 µg/g-rock), which was significantly reduced in oil-wet core samples. Alfazazi et al.^122^ also reported single-phase retention measurements on Indiana limestone outcrop samples with an average permeability of 150 mD. The experiments were conducted at 25 and 90℃ on separate core samples. Polymer retention was found to be 340 µg/g-rock at 25℃ and 194 µg/g-rock at 90℃. Skauge et al.^212^ also investigated polymer retention in carbonate rock samples with permeabilities ranging from 23 to 324 mD. The study used an ATBS-based polymer in high-salinity formation water of 180,000 ppm TDS. They reported low retention values and further discussed that polymer retention in the presence of oil was significantly reduced. They also showed that permeability variation has less impact on polymer retention in weakly oil-wet core samples than in the cores without oil. However, the authors highlighted that the results from their work were specific to a particular type of polymer and a single carbonate rock type. Hence, the result may vary with rock, type of polymer, and wettability conditions.

Seright et al.^32^ studied the stability and transport of different polymers, including ATBS-based polymers, in carbonate reservoir samples with moderate permeabilities (24–153 mD). They conducted their coreflooding experiments under anaerobic conditions and at a high temperature of 99℃ and salinity of 68,975 ppm in the absence of oil. The polymer retention values reported are between 200 and 911 µg/g-rock for polymer concentrations between 4,100 and 4,400 ppm. They suggested that the high retention could be due to the high polymer concentration and lack of oil in the core samples used. Moreover, no plugging was observed with ATBS-based polymer, even though the retention was relatively high. Wang et al.^225^ conducted dynamic retention experiments on carbonate reservoir cores at varied wettability conditions (in the presence and absence of oil). They utilized a sulfonated polyacrylamide polymer capable of withstanding high temperatures and salinities. Their experiments revealed low retention values (20.9–60.8 µg/g-rock). The polymer retention in the presence of residual oil was significantly lower than in the absence of oil. Wang et al. also reported results from different wettability conditions (including water-wet, oil-wet, and intermediate-wet conditions). Alfazazi et al.^103^ conducted polymer retention studies in the presence and absence of oil using Indiana limestone outcrop samples in a high salinity of 243,000 ppm TDS and a moderate temperature of 50℃. Additionally, high permeability samples (in the range of 150 mD) were used in their work. The authors reported low polymer retention values in single-phase experiments between 40–50 µg/g-rock. A significant reduction was observed in wettability-restored core samples.

Song et al.^137^ recently showed polymer transport in low-permeability carbonate rocks with permeability below 50 mD. A retention value less than 10 µg/g-rock was reported in 1ft Edward Yellow rock samples using a viscoelastic-sulfonated polymer, AN132. The authors mentioned that the low retention value could partially be due to the repeated filtration of the injected polymer solution through 1.2, 0.8, and 0.45 µm filters. They suggested continuous filtration removes the large macromolecules in the polymer solution. More interestingly, they also found that retention of the viscoelastic-sulfonated polymer increased slightly in the presence of residual oil. On the other hand, they observed high retention values (between 150 and 230 µg/g-rock) with one of the classical HPAM polymers, FP3130s. Sebastian et al.^233^ used ATBS-based polymer to carry out both static and dynamic retention measurements at 25℃ using different make-up water salinity ranging from 425–167,114 ppm. The make-up brine was prepared by dilutions of seawater (42,507 ppm) and formation water (167,144 ppm). In addition, some of the experiments were conducted in the presence of crude oil. They reported that by reducing the salinity of the make-up water, the polymer concentration required for a specific project can be optimized. The polymer in the diluted aqueous phase also resulted in a low adsorption value of 25 µg/g-rock. On the contrary, retention values between 47 and 56 µg/g-rock were reported in the formation water and seawater. A further reduction of retention was observed in the presence of oil.

### Injectivity of viscoelastic polymers

As previously mentioned, the primary purpose of polymers utilized in tertiary oil recovery is to control the injectant mobility and conformance by raising the viscosity and limiting the effective permeability of the aqueous phase. At high shear rate areas close to the wellbores, the dilatant behavior of viscoelastic polymers significantly increases the viscosity of a polymer solution, improving and boosting oil recovery efficiency. However, the extreme pressure buildup in the vicinity of the injection wells, related to viscoelastic flow and polymer mechanical degradation, severely limits the polymer injectivity. The injectivity refers to the injection flow rate divided by the pressure difference between injection pressure and average reservoir pressure^243^.

Injectivity is one of the primary considerations for effective polymer flooding applications. Therefore, the polymer solution should ideally have appropriate injectivity and maintain the desired solution viscosity at the same time^244^. Inadequate injectivity during viscoelastic polymer flooding may have unfavorable impacts, including slow propagation of injection fluid front, delayed oil production from flooded patterns, and voidage issues^21,104^. It was suggested to keep a polymer injectivity loss of 0.5 to 0.9 in order to ensure a uniform chemical injection at a given pressure and reduce reservoir voidage^245^. Thus, it is crucial to minimize severe shear-thickening at the wellbore to provide acceptable injectivity and prevent mechanical degradation of polymers. Shearing polymers before an injection is the optimum solution that reduces the elasticity of viscoelastic polymers and narrows the dilatancy window by shifting the onset for shear-thickening behavior to higher rates^102^.

Furthermore, the injection of viscoelastic polymers at high rates leads injection pressures to rise and exceed the formation fracturing pressure, generating fractures in the wellbore vicinity. Introducing fractures during polymer injection may have a dual impact on polymer flooding efficiency. First, if fracture propagation is not controlled, it may result in fluid channeling and adversely affect polymer sweep and displacement efficiencies^21^. In the cases of uncontrolled fracturing, the detrimental effects can be mitigated by plugging the fractured zones or reducing the polymer solution viscosity or injection rate in order to induce the fractures to close naturally^199^. On the other hand, creating short fractures extending just 5 to 15 m from the wellbore may favor the polymer flooding process by enhancing injectivity and reducing polymer mechanical degradation^102,200,246^.

## Viscoelastic polymer flooding field applications

Polymer flooding is the most extensively used cEOR technique in field-scale projects, which may result in substantial oil recovery at a cheaper cost and for a broader range of reservoir conditions^247,248^. Considering the economics of this process, the overall additional costs of polymer flooding may include the expenses for laboratory testing and development of suitable products, the installation of the appropriate facilities required to mix and inject the polymer solution, and purchasing the polymer product itself^249^. The cost of polymer separately ranges from 1 to 4 U.S. dollars per incremental barrel of oil produced (for example, in Daqing, this value is 2.7 U.S. dollars per barrel), resulting in a total cost for polymer flooding project between around 20 and 35 U.S. dollars per oil barrel^250,251^. Moreover, the primary objective of the polymer flooding approach is to boost oil recovery beyond the results of secondary waterflooding, resulting in more efficient use of present nonrenewable energy sources and a prolongation of the operational lifespan and profitability of mature oilfields^249^. The recovery efficiency achieved by polymer flooding is highly dependent on the amount of injected polymers, the mobility contrast between the aqueous and oleic phases, permeability distribution in a heterogeneous reservoir, and other parameters affecting polymer performance. In this regard, an increase in the amount of injected polymers typically boosts incremental oil recovery for a given project. Herein, the amount of injected polymers can be estimated by multiplying the polymer concentration in ppm and the injected polymer slug size in pore volumes (PV). However, it was shown that the incremental oil recovery becomes nearly insensitive to the injected polymer amount after the threshold value of 400 ppm**·**PV is exceeded^29^.

**Figure 15:**
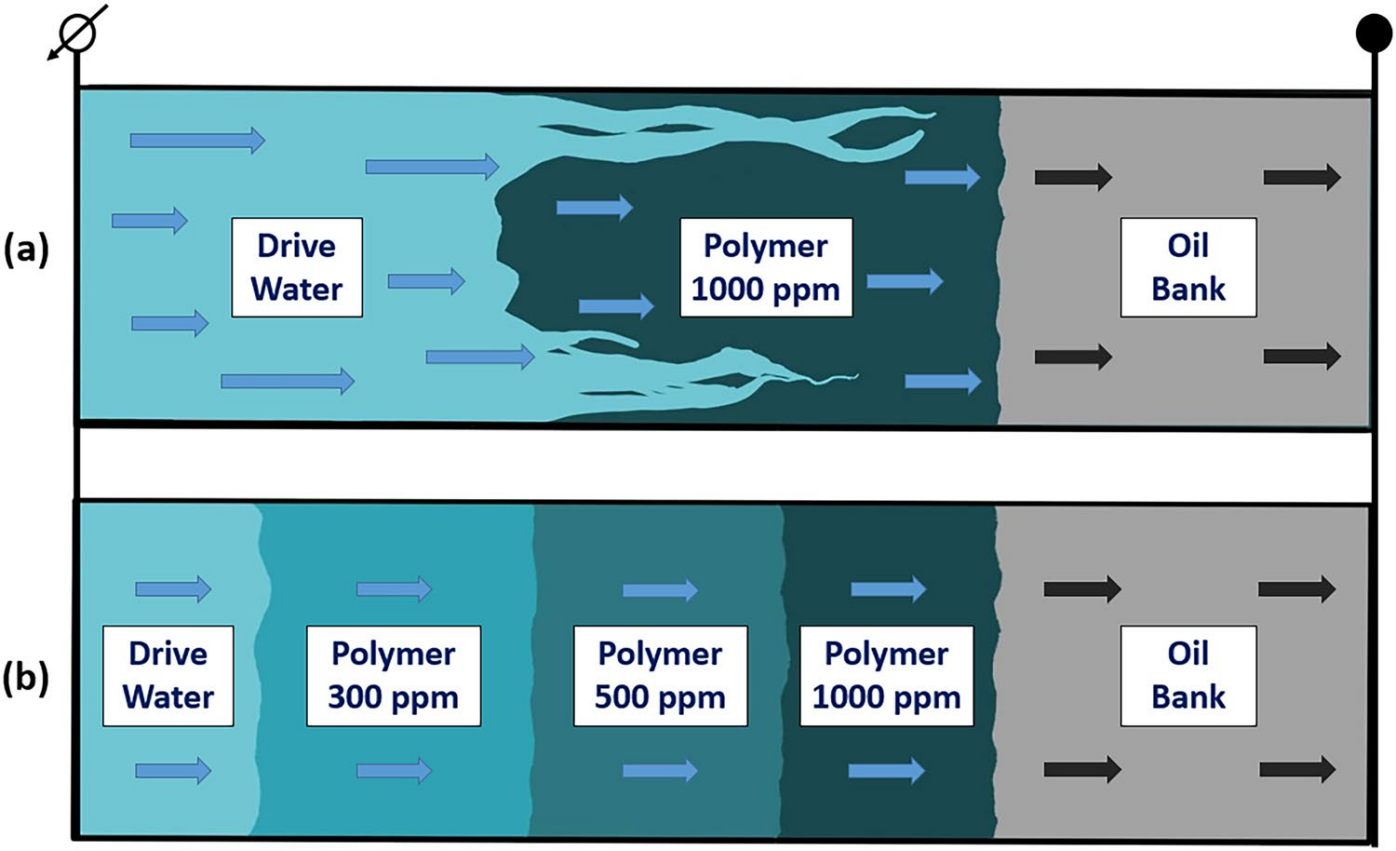
Grading polymer viscosity can mitigate viscous fingering of drive water following injected polymer solutions: (**a**) the polymer solution is injected as a single slug at a high concentration, and the subsequent drive water has considerable viscous fingering due to large viscosity contrast, (**b**) injecting the graded polymer banks by gradually tapering polymer concentration can reduce the amount of polymers needed for the project and significantly stabilize fluid fronts, mitigating viscous fingering of drive water.

Nevertheless, a low-concentration polymer flood may yield the same additional oil recovery as a higher-concentration polymer flood if the total amount of injected polymers is the same^252^. Moreover, grading polymer viscosity by gradually decreasing the injected polymer concentration in order to reduce viscous fingering after polymer injection is a frequently implemented strategy in field applications (Fig. 15)^199,253^. Although the tapered scheme mitigates mobility issues of drive water and reduces the amount of polymers required for a project, the constant high concentration scheme, on the other hand, may decrease the injection time for a polymer flooding technique, simplify the operation and reduce the overall cost^29^.

Furthermore, the molecular weight of the polymer is another crucial factor in polymer flooding projects. Greater M_w_ polymers often result in significant permeability reduction, improved viscosifying power, and increased incremental oil recovery. Nonetheless, this value should be chosen with care, taking into account formation permeability and pore-throat size distribution. Moreover, implementing polymer flooding technology in slightly heterogeneous reservoirs containing low to medium-viscosity crude oil is generally practical and highly efficient. Also, viscoelastic polymer injection may also be used in the thin heavy-oil reservoir, as thermal injection would be ineffective owing to heat losses^254^. Higher oil prices, reasonable polymer costs, increased use of horizontal wells, and careful injection control above the formation parting pressure can render polymer flooding more applicable in reservoirs with viscous oils^255,256^. However, polymer flooding may have certain limits in such reservoirs. For instance, it is impossible to attain a mobility ratio of one by injecting very viscous polymer solutions in the field, as injectivity concerns may increase project costs. Seright^256^ reported that in scenarios where there is no crossflow between layers, the primary advantage of polymer flooding in a two-layer reservoir with 1,000-cP oil is achieved by using a 10-cP polymer solution, while employing more viscous polymer solutions yields only a minor additional benefit. Conversely, in cases with free crossflow between layers, during polymer flooding in a two-layer reservoir with 1,000-cP oil, the preference shifts towards utilizing polymer solutions of higher viscosity ranging from 100 to 1,000 cP instead of a 10-cP polymer solution, provided there are no injectivity limitations. In other cases, horizontal injectors may be utilized to increase injectivity for this purpose^199^. Injecting polymer solution through horizontal wells might lead to an increase in heavy oil recovery of over 20%^257^. It was also reported that deploying the polymer flooding technology offshore as a primary or secondary injection mode might result in considerable enhancements to heavy oil recovery^258–260^. One instance of horizontal polymer injection in heavy oil reservoirs can be seen in the case of Milne Point Field's Viscous Oil Polymer Flood^261^. Milne Point Field initiated its first polymer injection pilots in 2018 on the North Slope of Alaska and made swift progress, achieving full-field polymer injection within four years. As of the end of 2021, they were injecting polymers at a rate of 32,000 bpd through 29 horizontal injection wells. The targeted reservoirs exhibit average permeabilities ranging from 100 to 1000 mD and in-situ oil viscosities ranging from 40 to 1300 cP. The concentration of injected polymer has fluctuated from the beginning of the operation, resulting in estimated in-situ end-point mobility ratios ranging from 0.8 to 2.0, with the lower mobility ratios typically introduced earlier during the flooding process. Moreover, both secondary and tertiary flood operations are being carried out in both greenfield and brownfield development areas, each yielding various but promising outcomes. Before the introduction of polymer flooding, there were issues with premature water breakthrough in the reservoir. This was primarily due to irregular spacing patterns, water fingering into the viscous oil, and the presence of loose sands that allowed water to bypass the intended pathways. This resulted in an inefficient waterflooding process and lower expected oil recovery rates. However, upon implementing polymer flooding, there was a notable improvement in pattern sweep efficiency, leading to a substantial increase in the anticipated oil recovery rates. The highest observed recovery rate stands at 27% of OOIP in a secondary polymer flood pattern, where the oil viscosity is 850 cP, and no water breakthrough has been observed thus far^261^.

Polymer flooding is a mature cEOR technology with more than 50 years of commercial application. Sheng et al.^29^ reported 733 polymer flooding projects in 24 countries worldwide. Accordingly, most of them were performed onshore, with just 8 offshore projects. Moreover, only about 104 projects were implemented in carbonate reservoirs. In the United States, polymer flooding commenced in the 60 s with the polymer injection in the Northeast Hallsville Crane unit to improve the injectant mobility and create the viscous barrier between the volatile oil zone and the large gas cap. Both economically and technically successful polymer flooding led to the incremental oil recovery of 3.19 million barrels in this field. Moreover, it was found that considerably better recovery was obtained in the areas where polymer flooding was implemented as a secondary recovery technique without water injection^262^. Subsequently, the number of active polymer projects in the USA has increased dramatically since 1978 (West Coyote, Vacuum, Eliasville Caddo, and other fields)^249^. Furthermore, numerous polymer flooding projects have also been performed in China, with an average additional oil recovery of around 9%^211^. However, the polymer flooding technique conducted in the Daqing Oil Field in China remains the most extensive and largest application of polymer flood worldwide^158^. Wang et al.^155^ reported that successful polymer flooding technology in the Daqing oilfield led to incremental oil recovery of more than 12% OOIP at operational expenses similar to waterflooding. Moreover, it was claimed that high recovery values were associated with viscoelastic polymers that improved volumetric sweep and displacement efficiencies.

Several field applications of viscoelastic synthetic polymers are summarized in Table 8^158,211,259^. According to the table, most of these polymer flooding field operations have been executed in sandstones under mild reservoir conditions, yielding on average about 10% additional oil recovery after waterflooding. Polymer flooding applications in carbonates are constrained due to the unfavorable parameters of this rock type. The reservoir heterogeneity and harsh conditions of elevated temperatures and salinities can considerably reduce the polymer flooding efficiency in most carbonates^27,191^. Thus, nearly no successful polymer field applications in harsh carbonates have been recorded^263^. However, the polymer injection project in the Eliasville Caddo Unit is one of the few examples of successful field-scale polymer flooding applications in carbonates. The tapered scheme was selected for the project, and the polymer was injected in four phases over three years. Ultimately, the polymer flooding yielded about 4.2% OOIP in this carbonate reservoir due to mild temperature conditions and a proper design of the polymer solution^264^.

**Table 8:** Summary of viscoelastic polymer flooding field applications.

No	Field	Rock and fluid properties					Polymer properties			IOR (%)	References
		Rock Type	$\overline{k }$(mD)	$\overline{T }$(°C)	Oil Viscosity In-situ (cP)	Connate Salinity (ppm)	C_p_ (ppm)	Solution Viscosity In-situ (cP)	Solution Salinity (ppm)		
1	Northeast Hallsville Crane (USA)	Limestone	50	109	0.09	N. A	250	N. A	N. A	18	Moore^262^
2	West Coyote (USA)	Sandstone	450	57	8	12,000	2,000	10–35	12,000	17	Shuler et al.^283^
3	Vacuum (USA)	Dolomite	17.3	38	0.88	169,000	50	1.5–5	387	1	Hovendick^252^
4	Eliasville Caddo (USA)	Limestone	11	46	3	165,000	630–1,500	5–35	1,200	4.2	Weiss^264^
5	Courtenay (France)	Sandstone	2,000	30	40	400	100–900	N. A	N. A	12	Putz et al.^284^
6	Captain (UK)	Sandstone	5,000	30.5	47–88	9,000–20,000	1250–4500	20–200	N. A	16	Pinnock and Clitheroe^285^, Poulsen et al.^286^
7	Xiaermen (China)	Fault-Block Sandstone	4,780	50	70	2,127	600–1,200	12–16.5	2,127	10	Deng et al.^287^, Sheng^211^
8	Daqing (China)	Sandstone	454	45	10	8,217.5	1,750–2,250	47.4	5,328	7–20	Wang et al.^155^, Guo et al.^288^
9	Marmul (Oman)	Sandstone	8,000–25,000	46	90	7,404	1,000	15	600	59 (total)	Sheng^211^
10	Xiaermen (China)	Sandstone	4,780	50	70	2,127	600–1,200	12–16.5	2,127	10	Sheng^211^
11	East Bodo (Canada)	Sandstone	1,000	50	1300	25,000–29,000	1,500	60	3,700	N. A	Sheng^211^
12	Abu Dhabi Oilfield (UAE)	Limestone	10–100; 1–10	120	N. A	200,000	2500	3.7	N. A	N. A	Masalmeh et al.^265^, Fabbri et al.^289^
13	Dalia (Angola)	Sandstone	> 1,000	50	1–10	120,000	900	3–6	52,000	7	Morel et al.^290^
14	Tambaredjo (Suriname)	Sandstone	4,000	38	400–600	2,500–5,000	1,000	50	400–500	11	Delamaide et al.^291^
15	Mangala (India)	Sandstone	200–20,000	65	15	N. A	500–2,000	2.5–30	5,267	7	Mehta et al.^292^
16	Shengli (China)	Sandstone	1,800	71	46.3	5,923	1,500	20.5	5,727	20.6	Guo et al.^288^
17	Xinjiang (China)	Conglomerate	189	34.3	5.13	28,868	1,000–1,200	> 60	7,031	12.1	Guo et al.^288^
18	Chengdau (China)	Sandstone	1,397	65	30–70	N. A	3000	> 25	N. A	11.6	Gathier et al.^293^
19	Milne Point Field (USA)	Sandstone	100–1000	20–32	40–1300	2,000–3,000	N. A	≈300–800	2,000–3,000	27	Edwards et al.^261^

Furthermore, novel polymers withstanding the extreme conditions prevailing in carbonates have been introduced lately. The new polymers include multiple monomers, such as N-vinylpyrrolidone (NVP) and Sodium Acrylamido Tert-Butyl Sulfonate (ATBS), preventing chemical and thermal degradation. ATBS-rich polymers were recently employed in a polymer flooding project in an Abu Dhabi carbonate reservoir^40^. The polymers were injected into a highly stratified limestone reservoir composed of two primary bodies with permeability contrasts ranging from one to two orders of magnitude^265^. Rachapudi et al.^263^ revealed that the single-well injectivity test in this reservoir demonstrated excellent injectivity and viscosity performance of novel polymers at a desired concentration and rate. The study also included injection skid and design description, candidate well selection for the injectivity test, polymer solution quality control, and operational challenges experienced during the pilot.

## Conclusions

This paper comprehensively reviews the recent studies on viscoelastic polymer flooding in sandstones and carbonates. The summary of the study and the main conclusions derived are listed below:

- Recent research indicates that viscoelastic polymers may also impact recovery efficiency at the micro-scale, despite the conventional belief. The primary mechanisms for mobilizing residual oil by viscoelastic polymers are pulling, stripping off, and dragging the oil globules.
- The main factors controlling the level of polymer viscoelasticity are relaxation and residence times. However, there is no consensus on calculations of residence time. Moreover, it is reported that estimating oscillatory relaxation time might not be adequate to predict polymer viscoelasticity correctly, especially under high salinity conditions. For this purpose, measuring the extensional relaxation time might be more representative of porous media.
- The terms strain hardening and shear-thickening are frequently used in the literature as the thickening in extensional and shear fields, respectively. However, the primary difference between them appears while estimating the onset of thickening. It was found that the polymer thickening starts at considerably lower fluxes in the extensional field than in the shear field. Therefore, it is crucial to establish a good connection between these terms or adhere to one of them to appropriately represent viscoelastic polymer flow in porous media, where both shear and extensional forces occur.
- Various viscoelastic models computing polymer apparent viscosity are listed in the study. UVM and AT-VEM models cannot capture the mechanical degradation regime, although the latter does not need coreflooding experiments to model the shear-thickening region. On the contrary, Stavland’s model and E-UVM capture all regimes exhibited by viscoelastic polymers in porous media, including mechanical degradation. Moreover, E-UVM was found to be more practical in fitting the experimental data and controlling the onsets and degrees of the shear-thickening and mechanical degradation regimes. It also provides empirical correlations relating its fitting parameters to rock and fluid properties that can considerably reduce the need for coreflooding tests. However, these correlations might be further improved to increase their accuracy under broader experimental conditions.
- The effect of various factors on polymer viscoelasticity was summarized in the study. Polymer molecular weight and injection rate lead to more pronounced viscoelasticity. Moreover, the positive effect of polymer concentration and the degree of hydrolysis on viscoelasticity disappears after a critical point. On the contrary, high permeability and temperature impair polymer viscoelasticity. The effect of solution salinity and hardness was found inconclusive. Finally, residual oil saturation has a dual impact on polymer viscoelastic behavior.
- Although viscoelastic polymers have great potential in displacing residual oil from sandstones, their applicability in carbonates is limited due to harsh conditions. It is suggested to implement the hybrid technique combining polymer flooding with EWF or LSW to improve the efficiency of the process. Moreover, novel polymers with increased resistance to harsh conditions have been recently designed for carbonates. However, their viscoelastic nature and effect on residual oil saturation have yet to be clearly understood and require further investigation.

## Data Availability

The datasets used and/or analyzed during the current study are available from the corresponding author on reasonable request.

## List of symbols

$${A}_{1}$$, $${A}_{2}$$: Empirical constants
$C$: Shear correction factor
${C}_{p}$: Polymer concentration (ppm)
$d$: Characteristic time constant for degradation
$\Delta D$: Vertical distance between the datum and the position below (m)
$$De$$: Deborah number
${E}_{d}$: Microscopic displacement efficiency
${E}_{v}$: Macroscopic sweep efficiency
$$g$$: Gravitational constant (m/s^2^)
${G}^{\prime}, { G}^{{\prime}{\prime}}$: Storage and loss moduli, respectively (Pa)
$j$: Tuning parameter for degradation
$$k$$: Absolute permeability (D)
${k}_{{p}^{\prime}}$: Effective permeability of displacing phase (D)
${k}_{w}$: Effective aqueous phase permeability (D)
${m}_{1}$: Shear-thinning exponent
$${m}_{2}$$: Elongational exponent
${M}_{w}$: Polymer molecular weight (MDa)
${N}_{B}, {N}_{C}$: Bond number and capillary number, respectively
${N}_{ce}$: Extensional capillary number
${N}_{crt}$: Critical capillary number
${N}_{T},{N}_{v}$: Trapping number and viscoelasticity number, respectively
${({N}_{T})}_{c}$: Critical trapping number
${({N}_{T})}_{T}$: Total desaturation or ultimate trapping number
$\overline{n }$: Average power-law constant
${n}_{1}$: Shear-thinning index
${n}_{2}$: Shear-thickening (or strain hardening) index
${n}_{3}$: Mechanical degradation index
${P}_{p^{\prime}}$: Pressure of displacing phase (Pa)
${r}_{1}$: Relaxation time constant
${r}_{2}$: Inverse of elongation onset
${S}_{orw}$: Residual oil saturation after waterflooding, respectively
${S}_{org}, {S}_{orp}$: Residual oil saturation after glycerin and polymer flooding, respectively
${S}_{or}^{*}$: Normalized oil saturation
${S}_{or1},{S}_{or2}$: Residual oil saturations estimated by trapping and Deborah numbers, respectively
${S}_{or1}^{low},{S}_{or1}^{high}$: Residual oil saturations at critical and total desaturation trapping numbers, respectively
${S}_{or2}^{low},{S}_{or2}^{high}$: Residual oil saturations at low and high Deborah numbers, respectively
${S}_{w}$: Aqueous phase saturation
${T}_{1}$: Trapping fitting parameter
${T}_{2}$: Viscoelastic fitting parameter (usually taken as 0.3–0.5 in sandstones)
$$t$$: Residence time (s)
$${u}_{w}$$: Darcy’s velocity (m/s)
$$V$$: Interstitial velocity (m/s)
$x$: Constant in the viscoelastic models (usually being equal to 2)

## Greek letters

$\alpha$: Angle of flow with respect to positive x axis
$$\theta $$: Contact angle
${\lambda }_{1}$: Shear-thinning constant
${\lambda }_{2}$: Shear-thickening constant (usually being equal to 0.01)
${\lambda }_{3}$: Mechanical degradation constant
${\mu }_{app}$: Apparent viscosity (cP)
${\mu }_{max}$: Maximum elongational viscosity (cP)
${{\mu }_{0}, \mu }_{\infty }$: Zero-shear rate and infinity-shear rate viscosities, respectively (cP)
${\mu }_{pore}$: Pore-apparent viscosity (cP)
$$\dot{\varepsilon }$$: Strain rate (s^−^^1^)
${\rho }_{p,} {\rho }_{{p}^{\prime}}$: Densities of displaced and displacing phases, respectively (g/cm^3^)
${\sigma }_{{p}^{\prime}p}$: Interfacial tension between the displacing and displaced phases (mN/m)
$\tau$: CDC model parameter dependent on the pore size distribution of the rock
${\tau }_{0}$: Empirical constant in the relaxation time model
${\tau }_{ext}$: Extensional relaxation time (s)
$${\tau }_{r}$$: Relaxation time (s)
${\Phi }_{p^{\prime}}$: Flow potential of displacing phase (Pa)
$$\phi $$: Porosity
$$\dot{\gamma }$$: Shear rate (s^−^^1^)
$\dot{{\gamma }_{1}},\dot{{\gamma }_{2}},\dot{{\gamma }_{3}}$: Onsets for polymer shear-thinning, shear-thickening, and mechanical degradation, respectively (s)
$\upomega$: Angular frequency (rad/s)

## Abbreviations

AA: Sodium acrylate
AM: Acrylamide
AMPS: Acrylamido-2-methylpropane-sulfonate
ATBS: Sodium acrylamido tertiobutyl sulfonate
AT-VEM: Azad-trivedi viscoelastic model
CaBER: Capillary breakup extensional rheometer
CAC: Critical association concentration
CBHAP: Comb micro-block hydrophobic association polymer
CDC: Capillary desaturation curve
CEOR: Chemical enhanced oil recovery
CT: Computed-tomography
DSNP: Dispersed silica nanoparticles in polyacrylamide
DLS: Dynamic light scattering
DPR: Disproportionate permeability reduction
EDC: Elastic desaturation curve
EOR: Enhanced oil recovery
E-UVM: Extended unified viscoelastic model
EWF: Engineered waterflooding
EWPF: Engineered water-polymer flooding
FENE-P: Finitely extensible nonlinear elastic-peterlin
FiSER: Filament stretching extensional rheometer
GO: Graphene oxide
HAP: Hydrophobically associated polymers
HCPN: High molar mass clay polymer nanocomposites
HPAM: Partially hydrolyzed polyacrylamide
HSNP: Hydrophilic silica-nanoparticle
HTHS: High-temperature and high-salinity
IFT: Interfacial tension
LCPN: Low molar mass clay polymer nanocomposites
LSW: Low-salinity waterflooding
MICP: Mercury injection capillary pressure
MWD: Molecular weight distribution
NP: Nanoparticle
NVP: N-vinylpyrrolidone
OOIP: Original oil in place
PAM: Polyacrylamide
PV: Pore volume
SLE: Solid-liquid equilibrium
TDS: Total dissolved solid
UCM: Upper-convected Maxwell model
UVM: Unified viscoelastic model
